# Chirality Transfer via Orientational Order of Micellar Assemblies on Gold Nanocrystals

**DOI:** 10.1002/adma.72905

**Published:** 2026-03-27

**Authors:** Robin Girod, Kyle Van Gordon, Fahim Faraji, Mikhail Mychinko, Francisco Bevilacqua, Cem Sevik, Milorad V. Milošević, Luis M. Liz‐Marzán, Sara Bals

**Affiliations:** ^1^ EMAT University of Antwerp Antwerp Belgium; ^2^ NANOLight Center of Excellence University of Antwerp Antwerp Belgium; ^3^ CIC biomaGUNE Basque Research and Technology Alliance (BRTA) Donostia‐San Sebastián Spain; ^4^ COMMIT Department of Physics University of Antwerp Antwerp Belgium; ^5^ CINBIO Universidade De Vigo Vigo Spain; ^6^ Centro de Investigación Biomédica en Red Bioingeniería Biomateriales y Nanomedicina (CIBER‐BBN) Donostia‐San Sebastián Spain; ^7^ Ikerbasque Bilbao Spain

**Keywords:** chiral plasmonics, electron tomography, gold nanoparticles, micelle templating, orientational order, seeded growth

## Abstract

Chiral Au nanocrystals are promising materials for biosensing and therapeutic applications. However, how chirality emerges during their seed‐mediated synthesis remains unclear, leading to limited control over morphologies and chiroptical properties. Herein, it is shown that chiral growth can be mediated by orientational order of chiral micelles at Au surfaces. Quantitative 3D electron microscopy and molecular dynamics simulations reveal growth rules for this mechanism and demonstrate that worm‐like micelles register with preferential crystal directions at the surface of the seeds to template the growth of hierarchically chiral features. These features have a specific torsion‐orientation coupling, which explains how both the molecular chiral inducer and the seed crystal structure can act as stereoselective parameters. These analyses suggest a new role of surfactant assemblies in seed‐mediated synthesis, and uncover fundamental aspects of chirality transfer with implications for the rational synthesis of chiral and anisotropic nanostructures.

## Graphical abstract

A mechanism for chiral growth of Au nanocrystals mediated by orientational order of chiral micelles at seed surfaces is demonstrated. 3D electron microscopy and molecular dynamics simulations reveal how alignment of the micelles along crystal directions and torsion–orientation coupling govern chirality transfer, providing a framework to rationalize existing morphologies and guide the design of nanostructures with enhanced chiroptical responses.

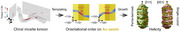

## 1 Introduction

Chiral Au nanocrystals, which have shapes that are not superimposable onto their mirror images, have the intriguing ability to exhibit circular dichroism (CD) originating from chiral localized surface plasmon resonances [1]. As a result, they feature strong differential extinction (absorption and scattering) between right‐handed and left‐handed polarized light, as measured by dissymmetry (*g*‐)factors typically orders of magnitude higher than those of chiral molecules [2, 3]. The well‐known sensitivity of surface plasmons to particle shape provides tunability in both magnitude and wavelength of the CD features, making these nanoparticles relevant for biosensing [4, 5], nanooptics [6, 7], and therapeutic applications [8, 9], among others.

To scale‐up the fabrication of nanoparticle enantiomers while retaining high uniformity, seed‐mediated colloidal synthesis has gathered increasing interest [3, 10, 11]. The general approach consists in the overgrowth of an achiral seed (including but not limited to cubes [10], octahedrons [12, 13], disks [6], or nanorods (NRs) [14]) in the presence of a chirality‐inducing agent that facilitates the removal of mirror planes in the produced shapes [3]. In particular, the recently coined micelle‐templated approach provides access to intricate morphologies with fine, 2–4 nm wide, surface features [11]. When these so‐called *wrinkles* are grown on Au NR seeds, they are known to be arranged quasi‐helically around the major axis of the particles and result in strong chiroptical activity in the visible and near‐infrared (NIR) spectral range [11, 15, 16, 17, 18, 19, 20]. Wrinkles emerge upon seeded‐growth in a surfactant mixture of cetyltrimethylammonium chloride (CTAC), which favors high deposition rates [11, 21, 22], and a molecular chirality inducer, typically 1,1′‐binaphthyl‐2,2′‐diamine (BINAMINE). BINAMINE and CTAC are thought to co‐assemble into worm‐like chiral micelles that subsequently coil around achiral NR seeds and ultimately template wrinkle growth (Figure S1) [11, 23]. With different combinations of seeds and ligands, the approach provides, in principle, access to an extensive library of morphologies with varying wrinkle spacing, width, and length, and thus continuously tunable chiroptical properties [24, 25]. Yet, despite ongoing efforts towards expanding the micelle‐templating toolbox, the precise synthesis of particles with predefined morphology and properties remains challenging.

A key bottleneck is the unclear mechanism of chirality emergence, which hinders the rationalization of the resulting structures. So far, it appears that the BINAMINE conformation influences the arrangement of the wrinkles. On single‐crystalline (SC) NR seeds, *S*‐BINAMINE yields right‐handed (*P*‐helical) wrinkled particles, whereas *R*‐BINAMINE yields left‐handed (*M*‐helical) ones [11]. It has been proposed that this reversal stems from a change in the helical sense of the chiral micelles, reversing in turn their coiling direction around the seeds [11]. However, other observations suggest more complex steps in the transfer of chirality. For example, seeded‐growth on penta‐twinned (PT) NR seeds yields morphologies with opposite handedness compared to those formed from SC seeds, even when the same BINAMINE enantiomer is used [16]. Moreover, the early stages of chiral growth have shown morphological and chiroptical discrepancies between SC and PT products [17]. This suggests an overlooked influence of the crystal habit or geometry of the seeds on the interactions of chiral micelles with Au surfaces.

To understand the emergence of chirality during micelle‐templated growth, we carried out a structural investigation of the chiral NRs at various stages of the growth process using electron tomography for 3D characterization [17, 26]. Our results reveal that a unified set of rules can explain the structure and morphology of the products grown from seeds with different crystal habits (SC or PT), so that chiral features exhibit order, consistent orientation, and consistent handedness in relation to the crystal surface onto which they grow. Supported by molecular dynamics (MD) simulations, we show that these observations originate primarily from the preferential alignment of chiral micelles along Au<100> and Au<111> crystal directions, i.e., that orientational order of surfactant assemblies can mediate seed‐mediated growth. Furthermore, we show that the templated features display hierarchical chirality and a specific torsion‐orientation coupling. As a result, chirality is controlled at the single‐feature scale–rather than at the NR scale–by the combined choice of molecular chiral inducer and crystalline surface of the seed. These findings show how chirality in nanocrystals can emerge from multiscale and collective effects, thereby disclosing a mechanism of surfactant‐directed growth that holds promise for the rational seed‐mediated synthesis of novel anisotropic structures.

## 2 Results and Discussion

### 2.1 Early‐growth Evolution of Wrinkled Particles

**FIGURE 1:**
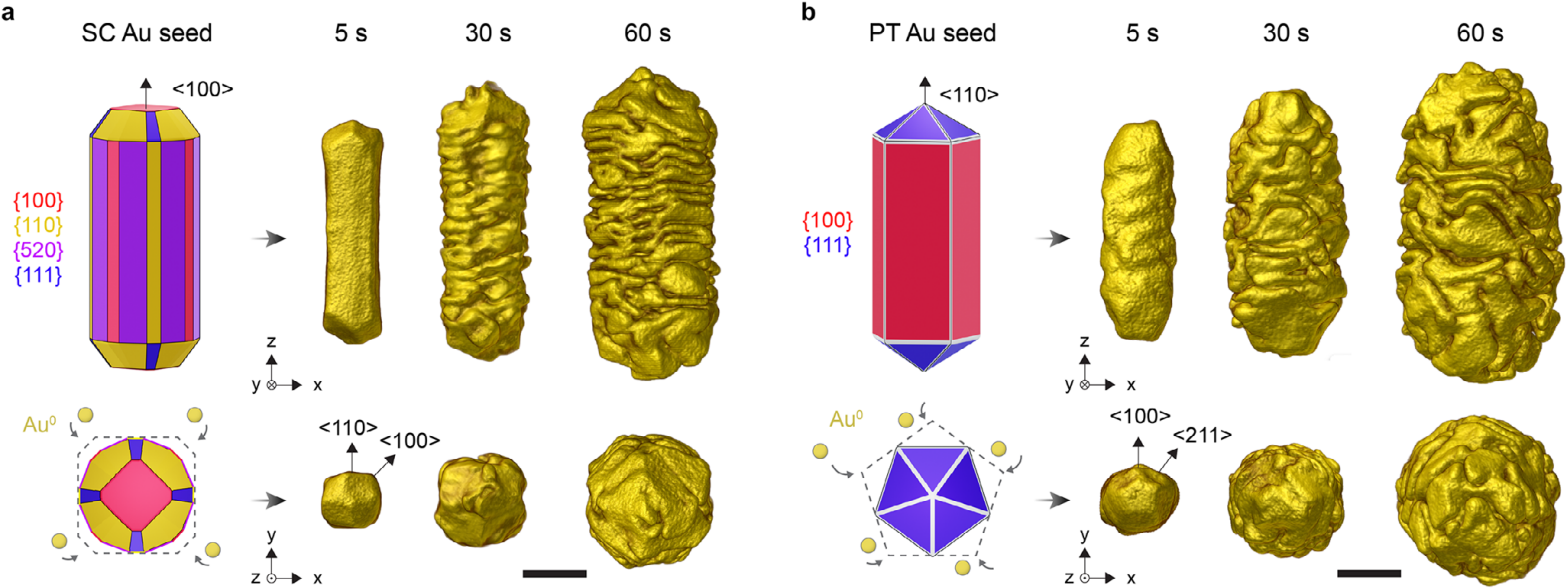
SC and PT seeds evolve wrinkles upon overgrowth in the presence of *S*‐BINAMINE. (a) Kinetic series of growth on SC NRs, and (b) PT NRs, showing the evolution from the seed (left, scheme, not to scale) into micelle‐templated products (right, surface rendering of representative particles at selected timepoints, reconstructed with electron tomography), in the presence of *S*‐BINAMINE. The bottom row shows the top views along the NRs' long axis, and (schematically) the preferential growth along <100> directions in the early stages. All scale bars are 30 nm.

To understand the emergence of chirality in wrinkled NRs, we investigated whether unifying patterns exist in the micelle‐templated growth of Au NRs with different crystal habits. We synthesized two series of samples by reducing HAuCl_4_ on SC or PT Au NR seeds using ascorbic acid as the reducing agent, in the presence of a mixture of CTAC and *S*‐ or *R*‐BINAMINE, and halting growth at selected time intervals (Figure 1a,b; Table S1) [17]. This was achieved through rapid addition of NaBH_4_, which causes fast reduction of remaining Au salt into spectator nanoparticles that can be separated via centrifugation. We have previously established that this approach allows to accurately retrieve the intermediate structures without significant influence of the NaBH_4_ addition on their properties [17], and the optical characterization of the series was reported elsewhere [17]. The structure of the initial seeds is well‐established: SC seeds have a combination of lateral surface facets of the {100}, {110}, and {520} families, are capped by {110} and {111} facets at the tips, and their long axis is oriented in a <100> direction [27, 28]; PT seeds have five {100} side surface facets with fivefold cyclic twin boundaries (TB) arranged around a <110> long axis, and are capped by {111} tip facets (Figure 1a,b) [29, 30, 31]. Figure 1 shows electron tomography reconstructions of representative intermediate products at selected timepoints.

We first aimed at understanding the apparent discrepancies between the two series in the early stages of growth. Specifically, the first SC intermediate product (after 5 s) showed 12 well‐defined surface facets and a quasi‐square cross‐section (Figure 1a). High‐resolution scanning transmission electron microscopy (STEM) images, as well as the measured angles between these facets indicate that they all belong to the {110} family (Figure S2a), which is in agreement with previous observations 15, 17, 24]. The products grown on PT seeds had less defined morphologies, and the inception of surface corrugation was already visible at 5 s after injection of the growth solution. Furthermore, sharp ridges and corners at the ends of the NRs resulted in fivefold symmetric tips that remained visible until ∼30 s of growth (Figure 1b). To identify their orientation, we performed electron tomography at atomic resolution on the tip of a wrinkled PT NR at 20 s of growth, when the ridges were already well developed (Figure S2b). The reconstruction shows that these ridges grew on the {100} side facets of the PT seeds. Accordingly, low angle annular dark field (LAADF)‐STEM electron tomography reconstructions, which provide diffraction contrast [31, 32], confirmed that twin boundaries extend towards the new lateral facets of the PT intermediate (Figure S3). Despite the apparent discrepancies, the initial stages of growth can therefore be consistently explained by preferential growth along <100> directions and formation of kinetically controlled products (Figure 1; Figure S2c,d) [3, 22].

### 2.2 Order in Micelle‐templated Au NRs

**FIGURE 2:**
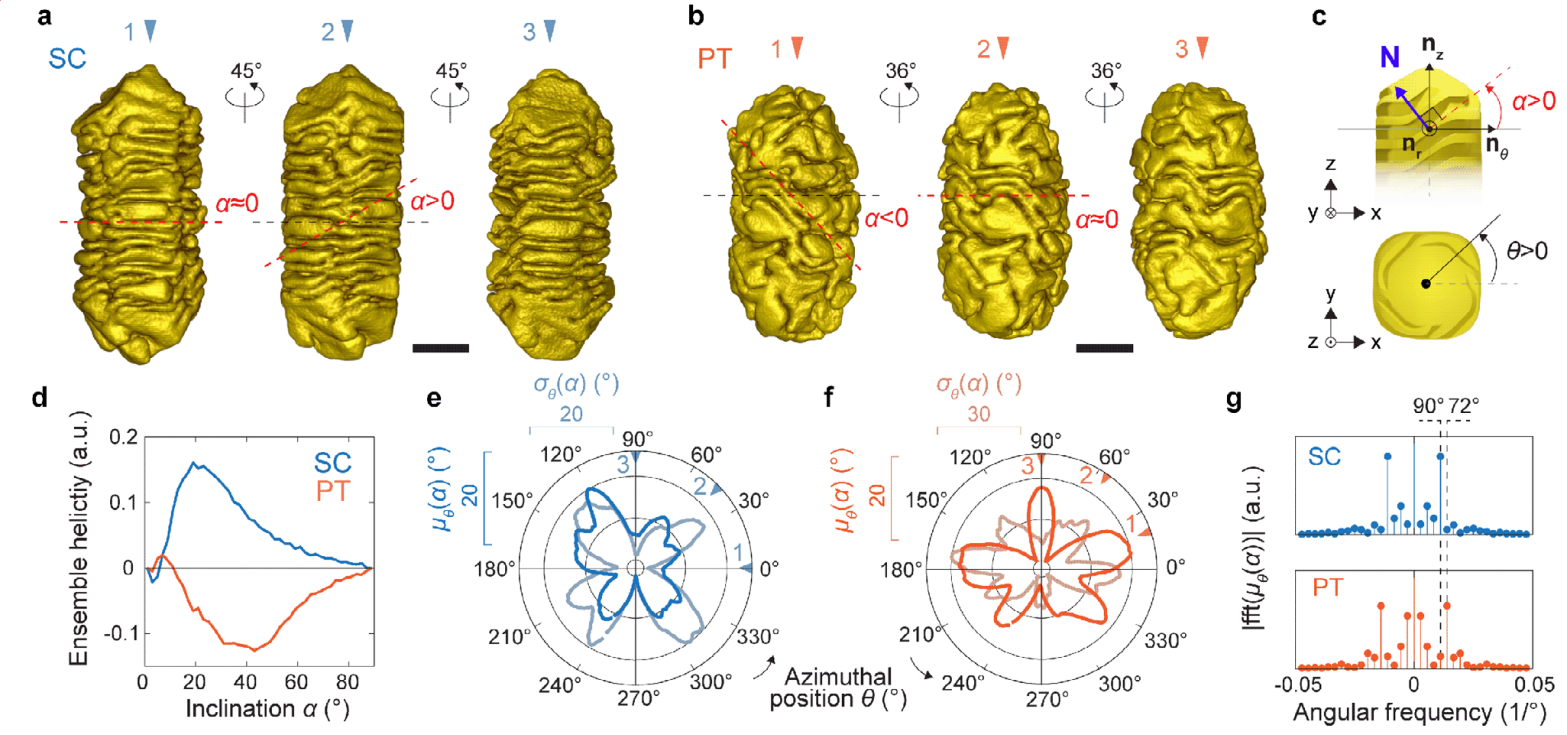
Morphology and order of micelle‐templated Au wrinkles. (a) Surface rendering of electron tomography reconstructions of a SC NR and (b) a PT NR after 60 s of growth in the presence of *S*‐BINAMINE. Scale bars are 30 nm. Dashed lines are included as guides showing the helical inclination angle *α* (red) of the features. (c) Depiction of the sign convention for *α* (red) and for the azimuthal angle *θ* (black). The scheme also shows the relationship between *α* and the **n_z_** and **n*_θ_*** components of the surface normal vector **N** (blue) in the (**n_r_**, **n*_θ_***, **n_z_**) local cylindrical frame. The inclination can therefore be calculated for every face of a triangulated mesh extracted from the tomography reconstructions (see Methods). (d) Helicity plot showing positive contributions from surface elements typically inclined at 12°–41° (at half maximum) across N = 10 wrinkled SC NRs, and negative contributions from surface elements typically inclined at 21°–61° in N = 10 wrinkled PT NRs. (e) The average (*µ_θ_*) and variance (*σ_θ_*) of the inclination angles calculated for the surface elements within a moving window of azimuthal positions *θ* shows oscillations at the surface of a SC NR and, (f) a PT NR after 60 s of growth in the presence of *S*‐BINAMINE. (g) Fourier analysis of *µ_θ_* (*α*) across N = 10 SC NRs and 10 PT NRs. Dashed lines are guides indicating the angular frequencies corresponding to 90° and 72°.

We then investigated how these intermediate structures further evolve during growth. On both types of seeds, well‐defined wrinkled features were eventually observed. The dimensions of these wrinkles were more homogeneous on SC products, but with close modal thicknesses on either seed type (SC versus PT products at 60 s of growth, 3.5 versus 4.2 nm), and a similar groove width of approximately 3 nm (Figure S4, and Table S2). These observations suggest a closely related growth process. In addition, both SC and PT products featured areas with non‐zero helical inclination *α* (Figure 2a,b), defined by analogy with the helix angle (Figure 2c). The typical inclination angle appeared positive on SC products (Figure 2a) but negative on PT products (Figure 2b). This difference was confirmed by measuring the helicity from the 3D reconstructions of SC and PT NRs across a range of growth times and synthesis conditions (see Methods and Table S1). Helicity quantifies the handedness of helical surfaces as a function of the inclination angle [17, 33, 34]. Ensemble plots (Figure 2d) show that chiral SC NRs grown in the presence of *S*‐BINAMINE featured positive helicity with maximum contribution from positive inclinations ∼12°–41° (as measured at the half maximum). For wrinkled NRs grown from PT seeds, helicity was negative, with maximum contributions from negative inclinations at ∼21–64°. Thus, products grown from SC NR seeds in the presence of *S‐*BINAMINE were right‐handed (*P‐*helical) whereas, products from PT NRs seeds were left‐handed (*M‐*helical) and featured overall steeper wrinkles. It is noteworthy that we have previously shown that helicity is a strong predictor of the sign and magnitude of the chiroptical response in helical NRs and that indeed, these SC and PT NRs were experimentally measured to have CD spectra with reversed signs [17].

Further observation of the wrinkle pattern showed that areas with non‐zero inclination periodically alternated with areas of approximately null inclination around both SC and PT products (Figure 2a,b). To quantify these patterns, we measured *α* as a function of the azimuthal position *θ* around the NRs (Figure 2e,f, and Methods) [26, 33]. Typically, four domains with non‐zero inclination alternating with four domains without inclination were observed around SC particles, whereas for PT particles, five domains with non‐zero inclination could be distinguished (Figure 2b–e). Fourier analysis of the inclination patterns averaged over the sampled PT and SC NRs confirmed the presence of dominant frequency components at 72° and 90°, respectively (Figure 2g; Table S1). The inclination of wrinkles therefore oscillates with fourfold rotational symmetry around particles grown from SC seeds, and with fivefold symmetry around particles grown from PT seeds. As these symmetries are also found in the initial seeds [14, 35], this result confirms that an interplay exists between the structure of the seeds, or their geometry, and the growth of wrinkles.

**FIGURE 3:**
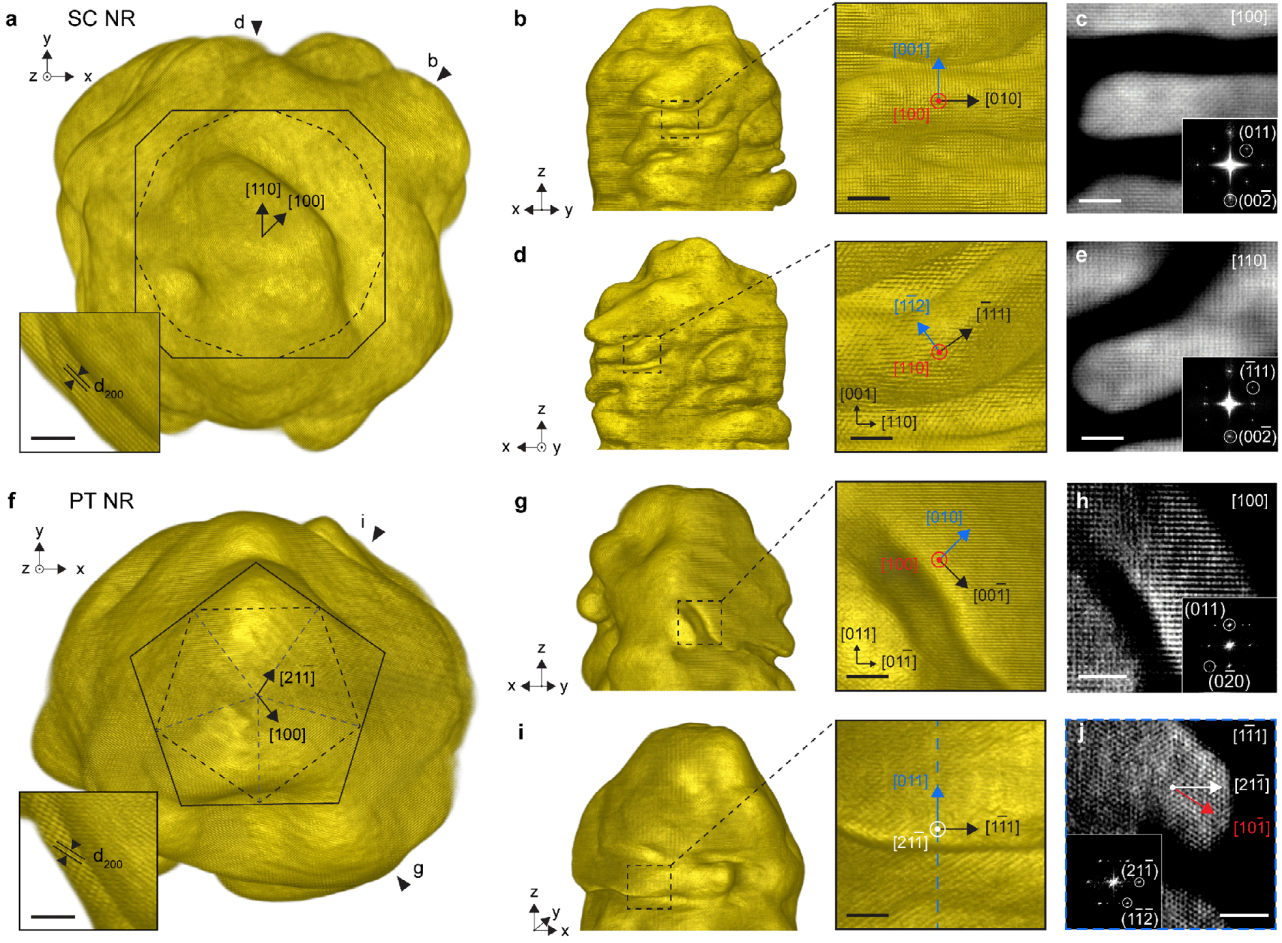
Crystal structure of BINAMINE‐CTAC micelle‐templated patterns from atomic resolution electron tomography. (a) Surface rendering of atomic resolution tomogram of a SC NR at 30 s of growth and (f) of a PT NR at 20 s of growth viewed down their long (*z*) axis. The insets are close‐ups showing {200} atomic planes, so that the orientation of the original seeds (dashed line) and of the intermediates (black line) can be retrieved. (b,d) surface rendering and, (c,e) slices through the tomogram of the SC NR with their corresponding FFT (inset). The viewing directions are indicated in **a** and face one of the lateral ridges of the SC intermediate in the [100] zone axis of the slice (b,c) and on of the lateral faces of the SC intermediate in the [110] zone axis (d,e). The multi‐color frame indicates the wrinkle alignment direction (black), the growth direction (red) and the binormal (blue), see also Figure S5. (g,i) Surface rendering and, (h,j) slices and FFT through the tomogram of the PT NR. The viewing directions are indicated in **f** and face a lateral ridge as seen at the tip of the particle and from the [100] zone axis (g) and a lateral facet (j). The slice in **j** is along the blue dashed line in **i** and shows a <111> zone axis, i.e., corresponds to a slice along a twin boundary. This wrinkle with null inclination did not grow in a direction perpendicular to the surface normal (which would be along <211>, white), but at a ∼30° angle to the normal, i.e., along <110> (red). Note that slices through the lattice and their FFTs are different from projection images, and that the size of the PT NR and its multiply twinned nature result in near‐atomic 3D reconstructions only. The FFT shows secondary peaks, which are artifacts from other under‐constrained domains of the twinned particle. All scale bars are 2 nm.

**FIGURE 4:**
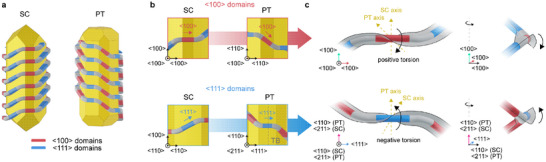
Chiral hierarchies in *S*‐BINAMINE‐CTAC micelle‐templated patterns. (a) Models of the patterns found in wrinkles grown in presence of *S‐*BINAMINE on SC and PT NRs. The underlying particle reflects the structure of the growth intermediates (Figure S2). In both SC and PT NRs, the wrinkles oscillate between areas aligned along <111> directions (blue), and areas aligned along <100> directions (red). (b) Close‐ups of the models showing the alignment of the domains as a function of the crystal structure of the NR seed. (c) Illustration of the torsion resulting from the wrinkle pattern shown as a rotation of the bending plane along the sub‐units with orientational order. Blue‐colored segments are aligned along <111>, red segments are aligned along <100>, gray segments are bending transitions. The gray plane depicts the bending plane along the length of the pattern; its direction of rotation gives the torsion sign and determines the local handedness.

To understand the origin of these oscillations, we carried out additional atomic resolution electron tomography measurements on wrinkled NRs. We found that the crystal structure of the wrinkles is equivalent, regardless of the seed type. On SC seeds, wrinkles with null inclination were typically aligned with and grew along <100> directions (Figure 3a–c; Figure S5). The inclined wrinkles on the other hand grew in <110> directions but their alignment was more variable, though often along <111> (Figure 3d,e). PT NRs were typically less resolved due to their size and multiple twins, but reconstructions nonetheless suggested similar wrinkle alignment and growth direction (Figure 3f–j). However, the inclined wrinkles were the ones aligned along <100>; those without inclination were aligned along <111>. The growth directions were also found to be along <100> and <110>. These observations were complemented by screening particles at early stages of growth with conventional electron tomography (Figure S6). On SC intermediate products, non‐inclined wrinkles appeared preferentially on the ridges, whereas inclined wrinkles correlated with lateral facets. On PT NRs, non‐inclined wrinkles typically correlated with lateral facets, whereas inclined ones were associated with lateral ridges. Given the structure of the intermediates (Figure S2), the position of the wrinkles and their typical angles (Figure 2d) are again compatible with preferential alignment along <100> and <111> directions, regardless of the seed (Figure 4a).

### 2.3 Multiscale Chirality and Handedness Selection

We then investigated whether the insights gathered so far could reconcile the opposite helical handedness in SC and PT wrinkled NRs. The wrinkles’ alignment along <100> and <111> directions sets the helical inclination of the wrinkles and thus the resulting helicity (Figure 4b). However, it is insufficient to rationalize handedness selection. Multiple crystal directions from the families along which alignment is observed are present on the facets of the seeds (e.g., [001] and [010] on Au(100), Figure S7). A selection rule must explain why one is preferred to the other. A mechanism of enantioselective growth of chiral facets could be proposed, as is typically proposed when amino acids are used as chiral inducers [6, 10, 14, 36], because the transitions from flat‐to‐inclined wrinkles contain high index microfacets or kinks, possibly chiral ones (Figure S8a). However, an overgrown helical NR could be obtained from enantiomerically pure facets of either *R* or *S* conformation (Figure S8b–d), so atomic‐level factors alone cannot explain the selection. Alternatively, the internal strain of twinned NRs could change the interactions of surfactant micelles with the Au seeds, or inhibit chiral evolution on twin boundaries as observed in pentagonal nanoprisms [35]. Yet, these effects cannot explain the selection of alignment along only one <100> direction because the directions from this family on Au(100) are at ± 45° from the major axis of the NRs (Figure S7) and should be subject to the same lattice strain [37, 38].

Instead, we found that unification of the growth on SC and PT NRs is possible under a hierarchical paradigm. This description builds upon the fact that *P‐* or *M‐*helical chirality at the ensemble NR‐level can be built from individual units (the wrinkle twists) with a different type of chirality. To show this, we use torsion as a wrinkle‐scale chirality descriptor. Torsion is a geometric property of space curves that quantifies the rotation of their bending plane along the centerline (Figure 4c; Figure S9) [39]. For example, an ideal right‐handed (resp. left‐handed) helix has constant positive (resp. negative) torsion (Figure S9). Here, the wrinkles periodically bend to align with <100> and <111> directions as they follow the surface of the NRs (Figure 4a,b). Accordingly, the bending plane of a wrinkle must rotate and counter‐rotate to accommodate the changes in direction, i.e., it must periodically exhibit positive and negative torsion (Figure 4c) [39, 40]. It then becomes apparent that the torsion sign follows the same pattern in the SC and PT NRs; for NRs grown with *S*‐BINAMINE, positive torsion happens in the <100> domains, whereas negative torsion happens in the <111> domains, regardless of the NR type (Figure 4c). The mirror patterns obtained with *R*‐BINAMINE have the opposite sequence; negative (resp. positive) torsion happens in the <100> (resp. <111>) domains regardless of the seed (Figure S10). The chirality of the wrinkles in micelle‐templated synthesis is therefore set by the specific combination of BINAMINE enantiomer and crystal direction along which they grow (Figure S10). This description shows that chirality in micelle‐templated NRs would be mediated by two interlinked effects: one setting the wrinkle‐scale handedness, and one setting their orientation with respect to the crystal surface, ultimately leading to opposite helical configurations on SC and PT NR seeds.

### 2.4 Orientational Order and Torsion of Chiral Surfactant Assemblies

**FIGURE 5:**
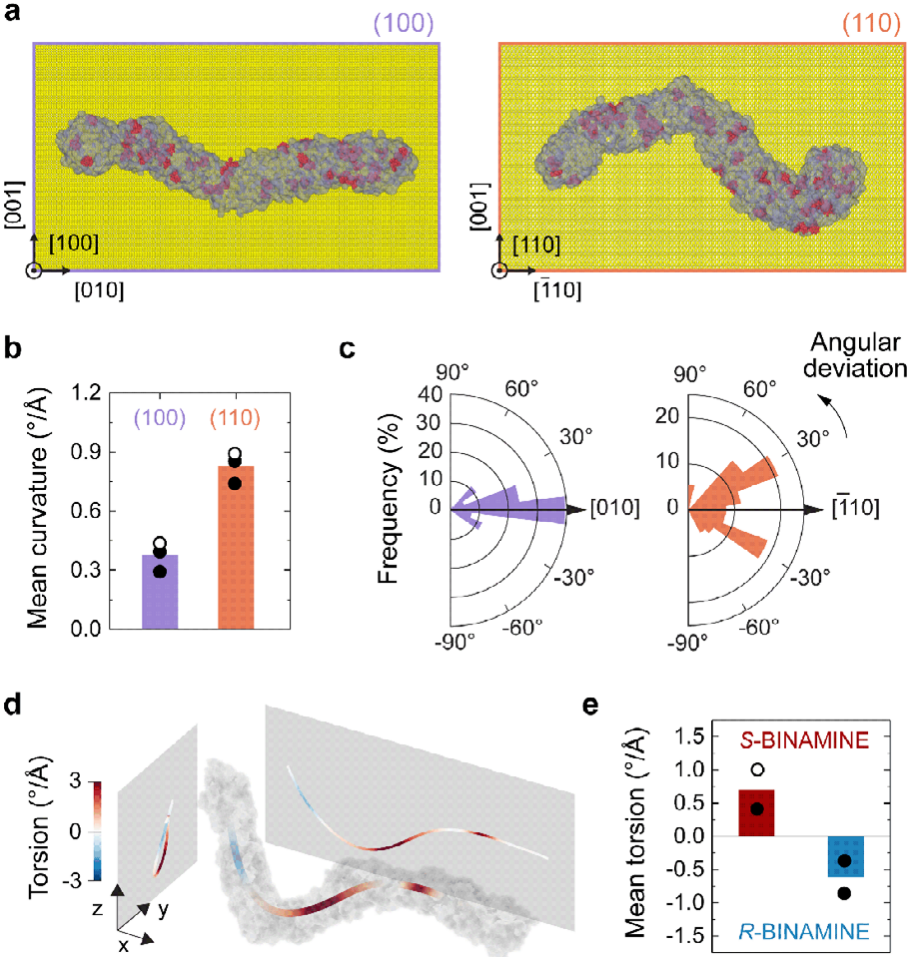
MD simulations of BINAMINE‐CTA^+^ micelles and their interactions with Au surfaces. (a) Molecular assemblies of *S‐*BINAMINE (red)—CTA^+^ (blue) on (100) (left) and (110) (right) Au surfaces (yellow). The simulations included a thin layer of water on the Au slabs (Methods), allowing mobility after adsorption. (b) Curvature of BINAMINE‐CTA^+^ assemblies on (100) and (110) surfaces. The curvature was computed with the Frenet‐Serret formulas and averaged along the arc‐length of the micelle center line (Methods, Figure S9–S14). Bars show the mean of N = 3 simulations on each surface, dots are the individual results, the open dots correspond to the simulations shown in **a**. The other simulations are shown in Figure S13. (c) Polar histograms of the angular deviation of N = 3 simulations of BINAMINE‐CTA^+^ assemblies on each surface. We define the angular deviation as the angle between a small element of the micelle center line projected onto the *xy* plane of the simulation frame and the **x** axis corresponding to the [010] or [‐110] direction (Figure S14). Histograms were computed for 1000 elements along the arc‐length of the fitted center line of each micelle. Histograms for individual micelles are shown in Figure S13. (d) Torsion along the center line of a molecular assembly of *S‐*BINAMINE and CTA^+^ (gray) stabilized in vacuum. Positive torsion indicates right‐handed twists, negative torsion left‐handed twists. (e) Mean torsion averaged along the center line of *S‐*BINAMINE—CTA^+^ (red) and *R‐*BINAMINE—CTA^+^ (blue) micelles in isolation. Bars show the mean values, dots the individual measurements form N = 2 independent simulations with each enantiomer (Figure S15), the open dot corresponds to the simulation in **d**.

To understand the origin of the configurations of wrinkled NRs, we further studied the molecular assemblies of BINAMINE and CTA^+^ that would template their growth. In liquid‐phase TEM images of the growth solution in graphene pockets, we found weakly‐contrasted, ordered, structures with a ∼6.3 nm spacing and threefold symmetry (Methods, Figure S11). These structures are consistent with the spacing of the grooves left at the surface of the overgrown chiral NRs, which was on average 6.4 nm (Figure S12). The spacing, symmetry, and order are remarkably similar to the behavior pure CTA^+^ micelles are known to exhibit on various inorganic crystal surfaces, including graphite and Au [41, 42, 43, 44]. Specifically, it has been consistently reported that (hemi)cylindrical micelles in 5–10 mm CTA^+^ solutions can form ordered assemblies with a 6.0 ± 0.5 nm periodicity and align along the <211> directions of Au(111) surfaces [42, 45, 46]. Despite the inclusion of BINAMINE and a higher CTA^+^ concentration in our synthesis conditions (20–50 m _m_ ), these observations suggest that also chiral micelles can exhibit orientational order. To explore their behavior along the crystal directions present on our seeds, we performed MD simulations of the adsorption of BINAMINE‐CTA^+^ micelles on Au(100) and Au(110) surfaces (Figure 5a; Figure S13). The micelles showed low curvature and high alignment along the [010] crystal direction on the (100) surface (Figure 5b,c; Figures S13 and S14). On Au(110), however, micelles formed zig‐zag patterns with angles around ±30°, which is compatible with the [‐111] and [‐11‐1] directions at ±35.3°. This suggests that the orientational order of CTA^+^‐rich chiral micelles along <100> and <111> directions is indeed possible, in agreement with the geometry of the wrinkles. Thus, our growth conditions appear suitable for the emergence of ordered molecular assemblies whose registration with specific crystal directions at the gold surface would explain the orientation of the wrinkles. We note that despite the single micelle simulated here, the energetically favored configuration of rodlike molecular assemblies is a parallel alignment [47], and thus the relatively high CTAC concentrations used in the synthesis should only reinforce collective orientation.

From the simulations, we also investigated the chirality of the micelles to understand the origin of wrinkle torsion. If unconstrained from the extended Au surface used in the model, the micelle developed the same torsion sign as wrinkles in the <100> domains of the corresponding growth products: *S*‐BINAMINE‐CTA^+^ micelles showed a net positive torsion in most places along their arc‐length (Figure 5d,e); *R*‐BINAMINE‐CTA^+^ micelles showed predominantly negative torsion (Figure 5e, Figure S15).

These results suggest that a complex interplay leads to the handedness selection for the overgrown NRs. Orientational order of CTAC micelles on gold likely arises from direction‐specific van der Waals interactions resulting in a torque that promotes alignment along low‐index crystal directions (Note S1) [43, 45]. Micellar torsion on the other hand, would be imparted by the BINAMINE molecules, possibly through a “sergeant‐and‐soldiers” mechanism [11, 48]. The van der Waals torque should compete with the bending and torsional forces of micelles [43, 45, 49], taking over on facets with <100> or <111> directions exposed. Furthermore, the fact that orientational order of wrinkles is clearly seen across the sharp ridges of the SC intermediate (Figure 1a; Figure S6b) shows that the micelles can follow these directions despite local curvature, in agreement with previous reports on rough surfaces [42]. This is key to the transfer of chirality to the wrinkles, because torsion is by definition an out‐of‐plate rotation that would vanish on a flat substrate. Thus, surface curvature is necessary for the micelles to still exhibit torsion once adsorbed. Nevertheless, wrinkle and micelle torsion correlate only along the <100> directions of the model proposed in Figure 4. This suggests that a different balance of forces is at play depending on the surface and alignment direction, or that a discontinued, non‐worm‐like micellar model should be considered (Note S2 and Figure S16) [50]. Either way, these simulations and measurements support a micellar origin for the order, alignment, and torsion of the fully grown wrinkles.

## 3 Conclusions

Our work demonstrates that unified growth rules govern the geometry and structure of micelle‐templated chiral NRs: (1) early growth is selective along <100> directions; (2) the subsequent wrinkle growth proceeds preferentially along low index crystal directions: <100> and <110>; (3) wrinkle alignment also follows preferential crystal directions, namely <100> and <111>; (4) their chirality is hierarchical and, at the wrinkle‐scale, depends on the BINAMINE enantiomer and the crystal direction along which they grow. These rules are consistent with all previously reported morphologies in micelle‐templated chiral Au NRs [11, 15, 16, 17], and provide guidance for future interpretation of these nanomaterials. In particular, the reversal of helical chirality in products grown on SC and PT NR seeds is explained by the difference in crystal directions at their surface. While evidenced on Au NRs, the rules may also apply to other Au seeds, in particular those exposing <100> and <111> crystal directions such as rhombic dodecahedrons or cuboids (Figure S17).

Furthermore, our results support a micelle‐templating mechanism in which both the micellar torsion, as set by the chiral co‐surfactant, and the orientational order of micelles on Au surfaces mediate the transfer of chirality. This suggests that the nanomechanics of surfactant assemblies can play an important role during colloidal growth and points to a large synthesis space to be explored towards fine control in chiral synthesis. For example, the competition between bending/torsion and orientational forces will be influenced by the choice of ligands and chiral co‐surfactant [25, 51]. Other co‐surfactants with anionic head (for insertion in the CTA^+^ palisade), affinity for both the gold surface and the micellar system, and chirality, may change the propensity of the micelle to twist [24]. The micelle curvature may also be tunable via the critical packing parameter of the surfactants [52], so long as they primarily interact with gold surfaces via van der Waals forces to allow for orientational order [53]. In that regard, the nature of the counterion is important. A more strongly adsorbed halide, such as Br^−^ will favor electrostatic interactions, which are less directional and will limit orientational order [42, 53]. For the specific goal of maximizing *g*‐factors in wrinkled NRs, we postulate that there should be an ideal facet size at which the balance of orientational forces and of those resulting from the micelle chirality would be optimal for the collective arrangement of micelles at the surface of the seeds. As a better wrinkle order is associated with a higher *g*‐factor [17], this would in turn provide a qualitative explanation for the volcano‐like trends in diameter versus *g‐*factor previously reported [11]. The fivefold rotational symmetry of the PT NRs also indicates these particles would feature more chiral centers than an SC NR of equivalent size, which appears promising towards high chiroptical responses if the wrinkle order can be optimized.

Finally, the finding that orientational order of micellar assemblies can occur not only on extended surfaces but also on faceted seeds and along previously unreported crystal directions holds promise for extending micelle‐templating beyond NRs, and towards novel chiral and anisotropic shape control at the nanoscale. We foresee that this new understanding of the morphologies produced with micelle‐templating will facilitate optimization of the (chir)optical properties for specific applications.

## 4 Methods

### 4.1 Chiral Au NRs

The NRs shown in the main figures of this work are original electron tomography reconstructions of samples synthesized for ref. [16]. Data used in the ensemble measurements and in the supporting information also include a reanalysis of reconstructions initially presented in refs. [16, 17]. The origin of the NRs, their main synthesis parameters (seed dimensions and crystal habit, BINAMINE enantiomer), and the figures they are used in are summarized in Table S1.

### 4.2 Conventional Electron Tomography

For specimen preparation, 1 µL droplets of the chiral Au NRs dispersions were placed on lacey‐carbon Cu grids (200 mesh, in‐house made) and left to dry. To prevent contamination during imaging, ligands were removed using the activated charcoal method [54].

Acquisitions for conventional electron tomography were performed using a probe‐corrected Titan Themis Z (Thermo Fisher Scientific, TFS) operated at 300 kV. HAADF‐STEM imaging was performed with a 50 pA probe current, 15–17 mrad convergence angle, and, typically, 46–215 mrad collection angle at the HAADF detector (115 mm camera length). To increase the visibility of twin boundaries in PT NRs, acquisition in LAADF‐STEM were also performed. Imaging conditions were similar, except a decreased collection angle to 23–142 mrad (230 mm camera length). The tilt‐series were aligned using a combination of cross‐correlation using an in‐house developed Matlab code and the vertical‐mass‐fluctuation Matlab code from the cSAXS toolbox [55]. 3D reconstructions were computed with the standard expectation‐maximization (EM) algorithm using the Astra toolbox 2.1 for Matlab [10, 11]. Non‐linear thickness effects, also known as cupping artefacts, were corrected following the assumption that the interior of homogeneous particles should have near‐constant intensity [56]. Isosurface renderings were created with Amira (v5.4.0). At least two particles were reconstructed for each sample analyzed.

### 4.3 Atomic Resolution Electron Tomography

Acquisitions for atomic resolution electron tomography were done using similar imaging conditions as conventional tomography, but using a 20–22 mrad convergence angle. Before acquisition, aberrations were corrected to achieve < 0.9 Å resolution on an Au cross‐grid. A Fischione 2040 dual‐axis holder was used, and the long axis of the NR was aligned with the rotation axis of the Titan stage. The angular range spanned at least [−75, +75]°. Acquisitions were done manually, in 2° increments overall and in 1° increments when approaching the <100>, <110>, and <111> zone axes. At each angle, five 2048 × 2028 px^2^ images were acquired with 0.3 µs dwell time.

For the reconstructions, complete tilt‐series were first assembled by aligning each angle‐specific series using a combination of cross‐correlation and optical flow, then averaging their intensities. The resulting images were restored with a convolutional neural network trained to correct noise and distortions [57]. The denoised tilt‐series were aligned with cross‐correlation, vertical‐mass‐fluctuation, and projection‐matching alignment as implemented in the cSAXS MATLAB toolbox [55]. 3D reconstructions were computed with the EM algorithm, again in Astra 2.1. Due to limited sample amounts, all particles studied with atomic resolution tomography had been grown with *R*‐BINAMINE, yielding, originally, left‐handed SC NRs and right‐handed PT NRs. To facilitate the discussion, the images in Figure 4 and Figure S5 have been mirrored.

### 4.4 Liquid‐phase TEM

Micellar assemblies were observed in graphene pockets for high‐resolution liquid‐phase TEM (LPTEM), following the methods for “encapsulation from the bottom” laid out in Pedrazo‐Tardajos et al. [58]. Briefly, ultraclean graphene grids were first manufactured in‐house from 200‐mesh Cu grids and single‐layer graphene (ACS materials, USA) [58]. For encapsulation, another piece of graphene was floated on water following dissolution of its Cu support and exchange of the etching solution. The graphene grid was placed at the bottom of the dish, and 10 µL of a 50 mm CTAC–2.5 mm BINAMINE solution, as used during chiral NR synthesis [17], was pipetted in between the grid and the floating graphene sheet, and water was then pipetted out until the sheet laid on top of the grid and eventually made contact. Imaging was performed at a dual corrected Titan Themis (TFS), operated at 80 kV in HRTEM mode. Dose rates were kept < 300 e^−^/nm^2^/s to prevent beam‐induce damage and gas nucleation in the liquid.

### 4.5 Morphological Analyses

Morphological analyses were based on triangulated surfaces meshes extracted from the conventional electron tomography reconstructions. Meshes were typically extracted using the Amira software, following binarization at a manually selected threshold. The procedure is equivalent to mesh extraction with the marching cube algorithm, followed by iterative simplification of the mesh until the number of faces was down to 400 000–500 000. After simplification, a final Laplacian smoothing pass was performed in Amira.

Analyses were primarily centered around inclination (α) measurements [17, 26, 33]. As shown in Figure 2, the α angle can be defined for each element of a surface mesh by measuring the Euler angles of the surface normal vector **N** in the local cylindrical basis (**n_z_**, **n_r_**, **n_θ_**). To visualize the azimuthal variation of wrinkle inclination, the average µ_θ_(α) and variance σ_θ_(α) of the inclination angles were computed within a moving azimuthal window of θ = 10° spanning the entire z‐length of the NRs. To investigate if the oscillations in µ_θ_ and σ_θ_ were consistent across particles, a Fourier analysis was conducted by taking the FFT of the averaged µ_θ_ curves across a range of SC (N = 10) and PT NRs (N = 10) with well‐defined wrinkles. These particles were representative of various synthesis conditions and growth time, as summarized in Table S1. Prior to averaging, the oscillation phase was matched for the different reconstructions by aligning the most prominent µ_θ_ peak.

Ensemble helicity measurements were conducted based on the previous definition of helicity [17]. Briefly, helicity is the integral of the inclination sign over a given surface, normalized to that surface:

(1) $$H_{total}≔\frac{\sum_{i}\mathrm{sign}\left(\alpha_{i}\right)S_{i}}{\sum_{i}S_{i}}$$

This also shows that helicity is the difference between the surface fraction that is right‐handed (positive inclination) and left‐handed (negative inclination), and it therefore a measure of helical handedness [17]. For ensemble measurements, helicity plots for individual particles were first calculated; following Equation (1), these are effectively histograms of the sign of the inclination angles, and are obtained by applying the helicity equation to inclination measurements within angular bins of 1°. These plots therefore show which inclination angles contribute most to the helicity measurements. The ensemble helicity plots were obtained by averaging the single‐particle plots of 10 and 10 SC and PT NRs, respectively.

### 4.6 Molecular Dynamics Simulations: Micelle Construction and Equilibration

Micelles were constructed by randomly distributing 50 *S*‐ or *R*‐BINAMINE and 500 cetyltrimethylammonium (CTA^+^) molecules within a cylindrical region of ∼440 Å in length and ∼40 Å in diameter. The S‐BINAMINE molecules were evenly placed along the cylinder axis. Bonded interactions (bonds, angles, and dihedrals) for both BINAMINE and CTA^+^ were described using the OPLS force field [59]. Nonbonded interactions were modeled with a 12–6 Lennard–Jones (LJ) potential for van der Waals forces and Coulombic terms for electrostatics. LJ interactions were truncated at 10 Å, and nonbonded parameters were taken from ref [60]. Cross‐interactions were determined using Lorentz–Berthelot mixing rules, and long‐range electrostatics were treated with the particle–particle particle–mesh (PPPM) method [61], with an accuracy of 1.0 × 10^−5^.

The initial micelle configuration was energy‐minimized using the conjugate gradient (CG) algorithm until the force tolerance reached 1.0 × 10^−4^ kcal mol^−1^ Å^−1^. The system was then equilibrated in the canonical (NVT) ensemble at 298 K using a Nosé–Hoover thermostat with a damping constant of 10 fs. Equations of motion were integrated with the velocity‐Verlet scheme using a timestep of 0.5 fs. All simulations were carried out with the LAMMPS package [62], and trajectories were visualized using OVITO [63].

### 4.7 Molecular Dynamics Simulations: Micelle Adsorption on Gold Substrates

To examine the adsorption behavior, a micelle was positioned above gold slabs exposing the (100), or (110) surfaces. The micelle, initially in its cylindrical (untwisted) conformation, was oriented at an (otherwise arbitrary) angle of 15° relative to the surface. This angle was chosen to prevent biasing the results by artificially initializing adsorption along a low‐index crystal direction. Each slab was covered with a ∼1.5 nm‐thick water layer, sufficient to allow micelle mobility while avoiding the additional computational expense of full solvation. The total simulation box dimensions were ∼600 × 250 × 80 Å^3^, with periodic boundary conditions applied in all directions.

Water molecules were modeled using the SPC/E model [64], and interactions among micelle, water, and gold atoms were described by Lennard–Jones potentials, with gold parameters taken from ref [65]. Water geometry was constrained with the SHAKE algorithm [66, 67] using a tolerance of 1.0 × 10^−6^ Å. The system was simulated in the NVT ensemble at 298 K with a Nosé–Hoover thermostat (damping constant: 10 fs). Gold atoms were fixed at their lattice sites throughout. To prevent water evaporation, a purely repulsive artificial “roof” was placed above the simulation cell, interacting only with water molecules via a weak LJ 12–6 potential (ε = 0.004 kcal mol^−1^, σ = 3.28 Å). Integration parameters for water were identical to those used for the micelle.

To explore the range of possible adsorption conformations, each simulation was repeated with varied initial conditions, including micelle–substrate distances. This approach allowed multiple adsorption pathways to be sampled and revealed a greater propensity for curved micelle configurations on the Au(110) surface compared with Au(100).

### 4.8 Simulation Analyses

The analyses of the simulated micelles were performed in MATLAB 2024b. Torsion, curvature, and angular deviation of the simulated micelles were measured from the centerline obtained by fitting a 3D spline to the atomic positions. The torsion, curvature, and Frenet–Serret frame were extracted along this curve with the robust‐Frenet Matlab software [40, 68]. The Frenet–Serret basis is an orthonormal basis defined at each point of the curve by the tangent vector **t**, a normal **n**, and a binormal **b**. **t** points in the direction of motion along the curve, while **n** is the derivative of the tangent with respect to the arclength (Figure S9). Their cross‐product yields **b**. These vectors define the curvature, which is the speed of rotation of **t** as it moves along the curve, and the torsion, the rotation of the osculating plane spanned by **n** and **t** (Figure S9) [39]. The angular deviation is defined as the local angle between the projection of the centerline on the *x‐y* plane of the simulation frame, and the given direction typically chosen to the *x*‐axis of the simulation frame (Figure S14). These analyses yield measurements for each point of the centerline so local variations can be visualized or plotted as histograms. To obtain summary metrics, the measurements were averaged along the length of the centerline.

## Author Contributions

R.G., L.L.M., and S.B. designed the research and conceived the project. R.G. and M.M. conducted TEM measurements and ET reconstructions. K.V.G. and F.B. synthesized the nanoparticles. F.F. conducted the MD simulations. C.S. and M.V.M. supervised the simulations and interpretation of the data. R.G. analyzed all the data and wrote the manuscript with L.L.M and S.B., and contributions from all authors.

## Funding

This work was funded in part by the European Research Council (ERC SYG 101166855, CHIRAL‐PRO to L.L.M and S.B). L.L.M. and S.B. acknowledge support by the EU through the HORIZON EUROPE MSCA SE project DELIGHT (10113111). R.G. acknowledges the support of a FWO postdoctoral fellowship under award 12A1V25N. The computational resources and services for this work were provided by the VSC (Flemish Supercomputer Center), funded by the FWO and the Flemish Government–department EWI.

## Conflicts of Interest

The authors declare no conflicts of interest.

## Supporting information




**Supporting File**: adma72905‐sup‐0001‐SuppMat.pdf.

## Data Availability

All data that support the findings of this study are present in the main text and the Supplementary Information. Raw data, measurements and original code developed for morphological analyses of electron tomography reconstructions and geometrical analyses of the simulated micelles are available at https://doi.org/10.5281/zenodo.19219177.
