# Supplementary material for: Chirality Transfer via Orientational Order of Micellar Assemblies on Gold Nanocrystals
Source: Adv Mater. 2026 Mar 27;38(24):e72905. doi: 10.1002/adma.72905 (PMC13113245; doi:10.1002/adma.72905)
Supplement: Supplementary file 1 — Supporting File: adma72905‐sup‐0001‐SuppMat.pdf. [file ADMA-38-e72905-s001.pdf]

Supplementary Information

Chirality Transfer through Orientational Order of Micellar Assemblies on Gold Nanocrystals

Robin Girod[1,2], Kyle Van Gordon[3], Fahim Faraji[2,4], Mikhail Mychinko[1,2], Francisco Bevilacqua[2], Cem Sevik[2,4], Milorad V. Milošević[2,4], Luis M. Liz-Marzán[3,5,6,7], Sara Bals[1,2*]*

[1]EMAT, University of Antwerp, B-2020 Antwerp, Belgium

[2]NANOLight Center of Excellence, University of Antwerp, B-2020 Antwerp, Belgium

[3]CIC biomaGUNE, Basque Research and Technology Alliance (BRTA), 20014 Donostia-San Sebastián, Spain

[4]Department of Physics, University of Antwerp, B-2020 Antwerp, Belgium

[5]Centro de Investigación Biomédica en Red, Bioingeniería, Biomateriales y Nanomedicina (CIBER-BBN), 20014 Donostia-San Sebastián, Spain

[6]Ikerbasque, 48009 Bilbao, Spain

[7]CINBIO, Universidade de Vigo, 36310 Vigo, Spain

*Corresponding authors' email: robin.girod@uantwerpen.be, sara.bals@uantwerpen.be

Content

Supplementary Note 1: Physical basis of orientational order 3
Supplementary Note 2: Handedness selection and orientational order 4
Supplementary Figures 5
Figure S1. Micelle-templated chiral growth 5
Figure S2. Structure of early growth products shows selective deposition along the <100> direction 6
Figure S3. Additional evidence for <100> growth on PT NR seeds 7
Figure S4. Morphology of wrinkles on SC and PT NRs 8
Figure S5. Additional slice and nomenclature for atomic scale wrinkle description 9
Figure S6. The inclination of wrinkles depends on their position around chiral NRs 10
Figure S7. Crystal orientations at the surface of wrinkled Au NRs 12
Figure S8. Possible high index (chiral) surfaces in micelle-templated wrinkles 13
Figure S9. Torsion and curvature of helices 15
Figure S10. Comparison of wrinkle torsion in NRs grown with *S*- or *R*-BINAMINE 16
Figure S11. Orientational order of BINAMINE-CTAC multimicellar assemblies in graphene liquid cells (GLC) 17
Figure S12. Distribution of the groove-to-groove distance on Au NRs 18
Figure S13. MD simulation of BINAMINE-CTA+ assemblies on Au 19
Figure S14. Geometric properties of micelles from MD simulations 20
Figure S15. MD simulations of isolated BINAMINE-CTA+ assembly 21
Figure S16. Discontinuous models of wrinkles and adsorbed micelles 22
Figure S17: Potential for orientational order of CTAC-BINAMINE micelles on common Au surfaces and seeds 23
Supplementary Tables 24
Table S1. Overview of NR synthesis 24
Table S2. Morphological measurements of wrinkles. 24
Supplementary References 25

Supplementary Note 1: Physical basis of orientational order

The physical basis of orientational order can be discussed in light of the literature on CTA^+ micelles on Au(111) (ref. [1–3]) and graphite.[2,4,5] These reports globally support that CTAC forms hemicylindrical micelles on both surfaces, i.e., that CTAC interacts primarily via the alkane tail.[2] According to DFT calculations by Saville *et al.*,[4] there is a small energy gap between the different orientations of an adsorbed CTAC molecule in such configuration. However, this energy difference is on the order of the thermal energy kT, and is unlikely to drive the collective alignment of micelles.

It has been proposed, instead, that orientational order arises from effects at the colloidal scale.[4,5] At this scale, the relative orientation between a rod (modelling a micelle) and a surface is determined by torques originating from various intermolecular forces, in particular anisotropic van der Waals interactions. Indeed, a crystalline surface can exhibit a direction-dependent dielectric response when the separation is in the order of nanometers (even for a uniaxial lattice such as graphite[6] and, supposedly, for an FCC crystal like gold too). This effect was shown to increase the probability of alignment perpendicular to the symmetry axes of the basal plane of graphite at short separation distances. Similarly, this suggests that the physical basis for alignment on gold, for example along <100> rather than <110> on Au(100) would be a different dielectric response along these directions translating into direction-specific van der Waals interactions.

It is noteworthy that gold surfaces can interact with both headgroup and tail of the surfactants. Orientational order is therefore modulated by the relative strength of alkane-gold vs. counterion-gold adsorption.[1] Specifically, a weakly adsorbing counterion is needed to favor van der Waals interactions over electrostatic ones. For example, CTAC and CTAOH display strong orientational order on both Au and graphite,[1,4] but CTAB does only on graphite presumably because the interactions with Au become dominated by the more strongly adsorbed Br^-.[2,7]

Supplementary Note 2: Handedness selection and orientational order

Our structural analysis and MD simulations show a correlation between the torsion of wrinkles along <100> domains and that of micelles in isolation, whereas the torsion along <111> domains is the opposite of that in isolation. This correlation is crucial to the handedness selection. Should the torsion and alignment alone be considered, there would be no reason for *S*-BINAMINE micelles (positive torsion in isolation, **Figure S13**) to yield *P*-helical PT NRs when a *M*-helical PT NR could still show <100> and <111> wrinkle alignment with periodically positive wrinkle torsion, albeit along <111> domains. The correlation therefore suggests that it is energetically favorable for the micelles to maintain their torsion in the <100> domains rather than in the <111> domains. This could arise from a faster adsorption on the {100} facets, or stronger alignment forces along <100> crystal directions. Orientational order would then start along <100>, proceed along <111> following the preferred torsion sign, and back along <100>. It is noteworthy that the alternating torsion sign only appears under a continuous, worm-like model.[8] Alternatively, the combination of van der Waals torque and torsion could overcome the scission energy of the micelles to create cap ends on either sides of the <100> domains,[9] which would then be free to follow the preferential micelle handedness without inversion of the torsion sign (**Figure S14**). This is experimentally consistent with the better definition of wrinkles in <100> domains, particularly in SC NRs.

Supplementary Figures

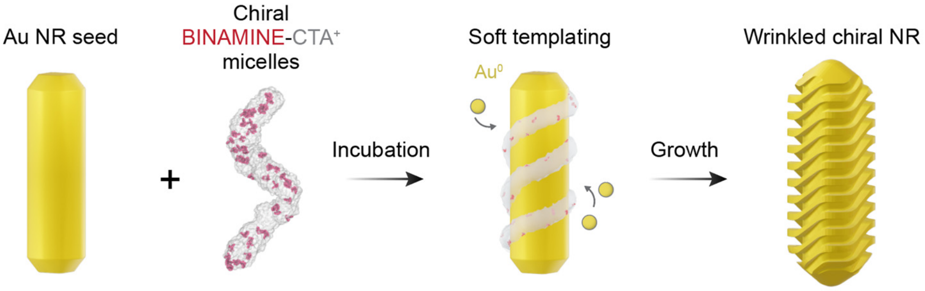

Figure S1. Micelle-templated chiral growth. Illustration of the micelle-templated, seed-meditated growth process to obtain wrinkled chiral Au NR.

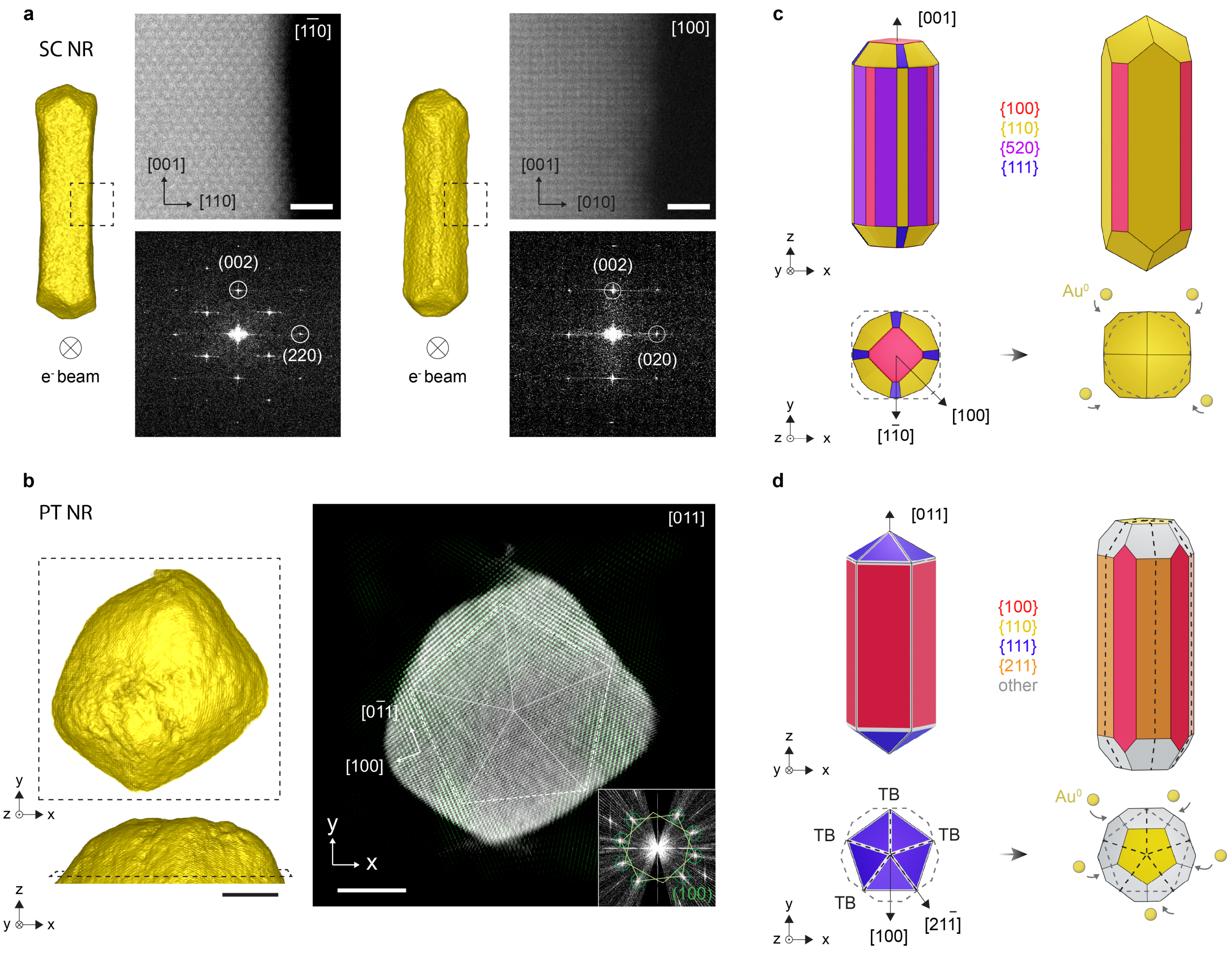

Figure S2. Structure of early growth products shows selective deposition along the <100> directions. **a**, Structural characterization of an intermediate product after 5 s of growth on a SC seed, shown in HAADF-STEM images. Left: image and corresponding FFT obtained along a <110> zone axis when a lateral facet was oriented to face the incident beam direction. Right: image and corresponding FFT obtained along a <100> zone axis when a lateral ridge was oriented to face the incident beam. The 3D renderings are based on an electron tomography reconstruction indicating the orientation and the typical imaging area (dashed box). The scale bars are 1 nm. **b**, Structural characterization of an intermediate product after 20 s of growth on a PT NR seed, shown in atomic resolution electron tomography. Left: surface rendering of the particle's tip. Right: cross sectional slice, obtained according to the dashed *xz* plane, perpendicular to the [011] zone axis. The inset shows the FFT of the image. Fringes with d_{100} are identified from a superimposed Fourier filtered image (green) and correspond to the original lateral facets of the seed. White dashed and continuous lines are guides representing the orientation of the original seed and twin planes, respectively. The scale bar is 5 nm. The inset shows the FFT of the image, dark green circles indicate the reflections that were kept in the Fourier filter, light green pentagons highlight the 5-fold symmetry of the structure. **c**, Models of the early changes during growth on SC and, **d**, PT NR seeds (not to scale). Gray dashed lines show the morphological evolution, black dashed lined denote the twin boundaries (TB). Note that the models are idealized and are more sharply facetted than real products to facilitate crystallographic discussion and show that the obtained structures are consistent with growth along <100> directions.

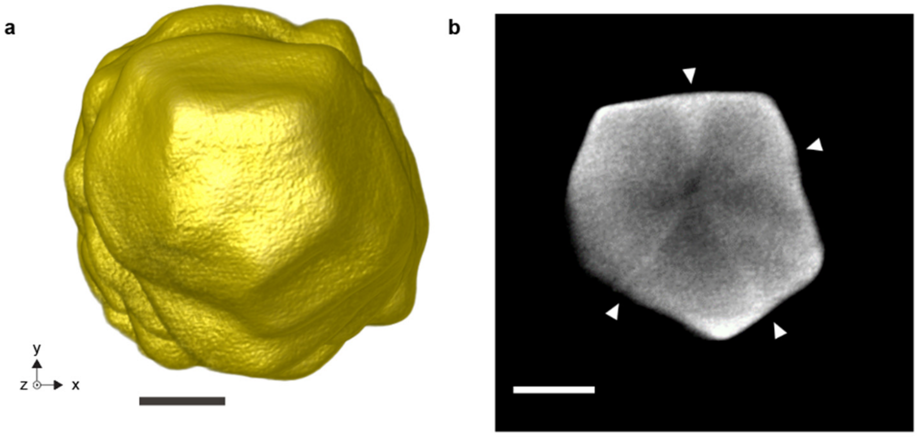

Figure S3. Additional evidence for <100> growth on PT NR seeds. a, Isosurface rendering and **b**, orthoslice through a tomographic reconstruction of a PT NR after 20 s of growth in presence of *S*-BINAMINE. Electron tomography was performed in LAADF-STEM condition to include diffraction contrast.[10] Conventional HAADF-STEM images result from electron incoherently scattered at high angles (typically, ≥ 50 mrad), which yields intensities varying with ~$tZ^{1.5\text{-}2}$ (t is the thickness, Z the atomic mass) and fulfills the projection requirement of tomography.[11] If a smaller collection angle is used however, diffracted electrons will also be collected when one of the crystalline grains is in zone axis. As a result, the interior intensities will not be a faithful representation of the material's density but will highlight the grain boundaries. The bright features thus correspond to twin boundaries, which are seen to reach the facets of the overgrown rod. The scale bars are 10 nm.

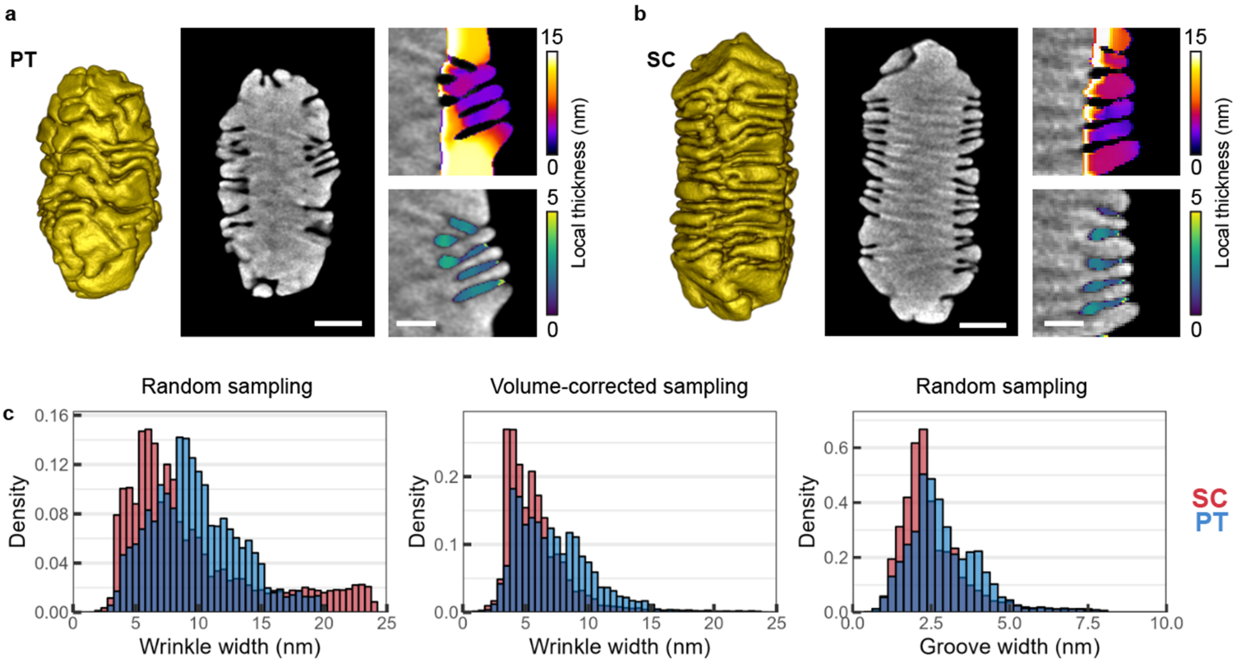

Figure S4. Morphology of wrinkles on SC and PT NRs. a, Surface rendering and orthoslice of an electron tomography reconstruction of a chiral Au NR after 60 s of growth in presence of *S*-BINAMINE on a PT, or **b**, a SC NR seed. Close-ups show typical results from measurements of the wrinkle thickness (top) and groove width (bottom). The results are obtained using local thickness measurements on the interior of the NR, respectively the exterior, and applying a cutoff value defining a wrinkle or a groove. The methods are further described in ref.[12] Scale bars are 25 nm in the orthoslices, 10 nm in the close-ups. The surface renders are displayed at the same scale. **c**, Histograms of the wrinkle and groove thickness. Each PT and SC distributions correspond to 10,000 points sampled in the local thickness maps of 2 representative NRs at 60 s of growth (total number of measurements per distribution N = 20,000). Typically, sampling the local thickness maps would be done randomly. Therefore, the probability that a given feature is sampled is proportional to its volume which yields, by default, a volume-weighted distribution (left). However, it may be considered that wrinkles are relatively well-defined and that a width distribution should sample each of them with equal probability (for example, that a small and a large wrinkle should each yield one measurement as might be done manually). We model this process by associating a volume-corrected sampling probability to the voxelated thickness map. Specifically, the sampling probability of a given voxel is set to be the inverse of the volume of a sphere whose diameter is the thickness at this voxel, yielding a volume-corrected distribution (center). This distribution makes apparent that the modal wrinkle width is close in SC and PT NRs, coherently with the fine wrinkles looking very similar in their respective 3D reconstructions. The SC distribution nonetheless remains narrower, confirming, along with the qualitative observations, that wrinkles were more homogeneous in this batch of micelle-templated SC NRs. The distribution of the width of the groove, assumed to correspond to the imprint of the templating micelle was however very similar for the two NR types, even with random (non-volume-corrected) sampling. This suggests that the same type of micelles participated in the templating mechanism. Summary statistics are in **Table S2**.

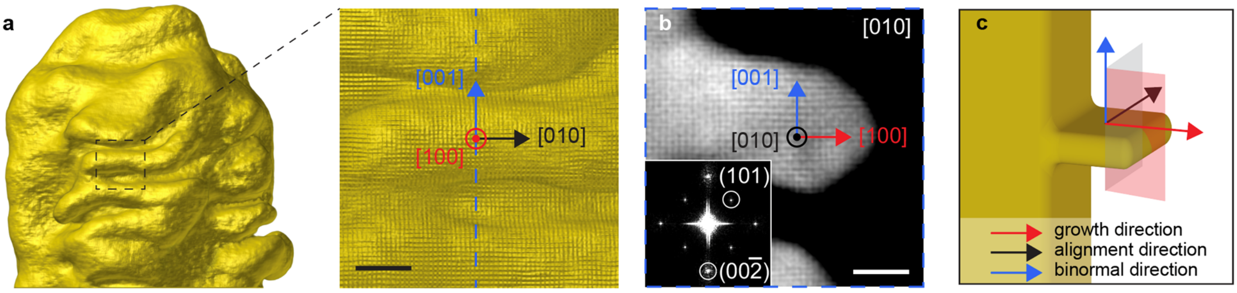

Figure S5. Additional slice and nomenclature for atomic scale wrinkle description. a, Surface rendering and, **b**, slice and FFT of the SC NR at 30 seconds of growth shown in **Figure 3** of the main text. **c**, Wrinkle depiction and nomenclature used here and in the main text. The alignment direction refers to the direction followed when moving along the long dimension of the wrinkle. The growth direction refers to the direction followed when moving along the medium dimension of the wrinkle. The binormal direction (blue) refers to the direction followed when moving along the short dimension of the wrinkle. Here, **b**, is a slice in the binormal-growth direction plane (blue dashes in **a**). In the main **Figure 3**, most slices show slices in the binormal-alignment direction plane.

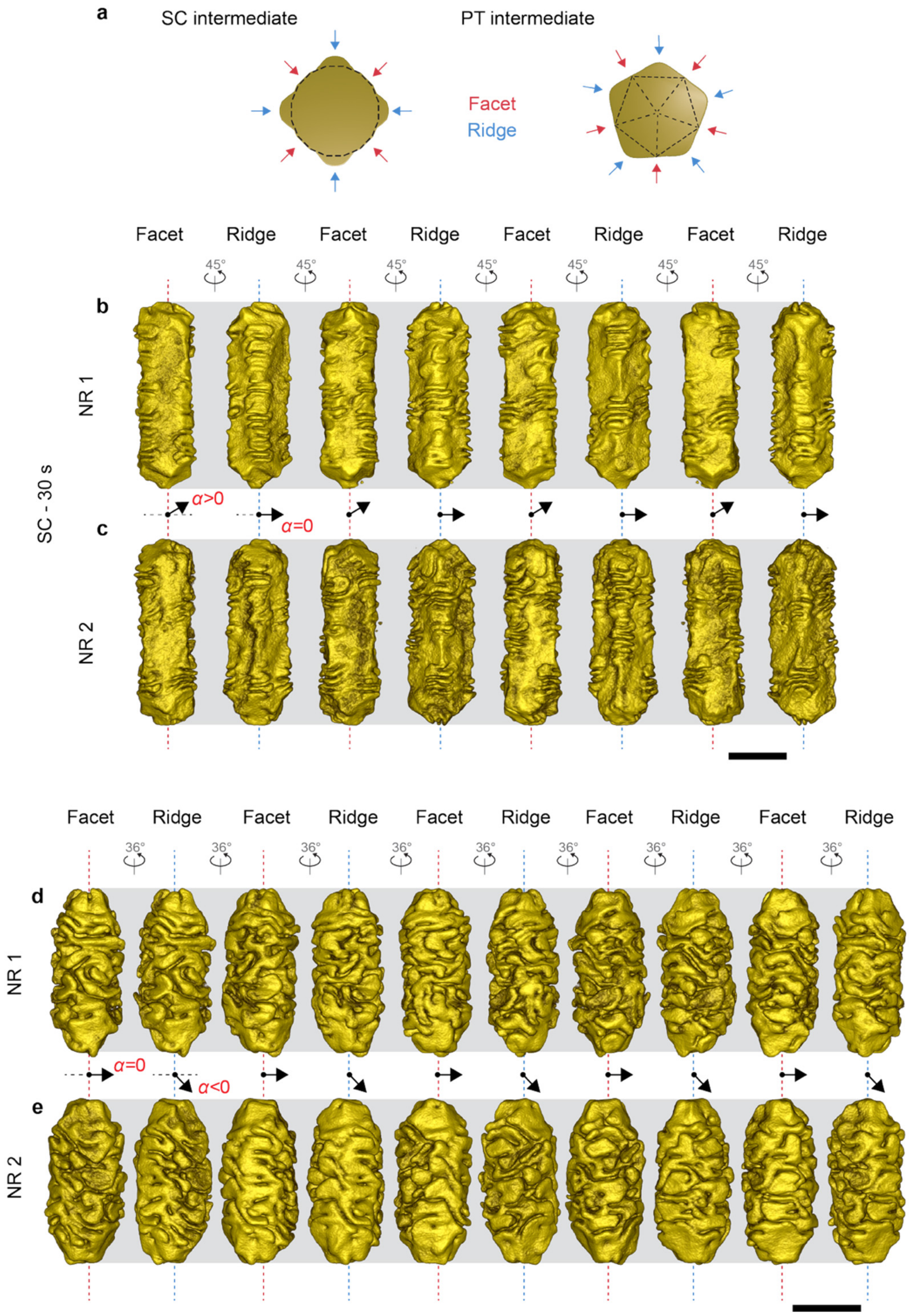

Figure S6. The inclination of wrinkles depends on their position around chiral NRs. a, Depiction of a cross-section along the long axis of micelle-templated growth intermediates from SC or PT NR seeds. Dashed lines show the structure of the initial seeds. **b, c**, Isosurface rendering from electron tomography reconstructions of SC and, **d, e**, PT NRs after 30 s of growth in presence of S-BINAMINE. Wrinkles without inclination angles were typically seen on the ridges of the SC intermediate, but on the facets of the PT one. Wrinkles with inclination were seen on the facets of the SC intermediate, respectively on the ridges of the PT ones. The black arrows are guides indicating flat inclination ($\alpha \approx 0°$), a 35.3° positive inclination α corresponding to <111> directions, and a -45° negative inclination corresponding to <100> directions. Additional discrepancies between SC and PT products include a

more advanced development stage on PT seeds, that wrinkles were less developed and continuous on SC NRs, and that the wrinkles on PT NRs appear more disordered. The scale bars are 50 nm.

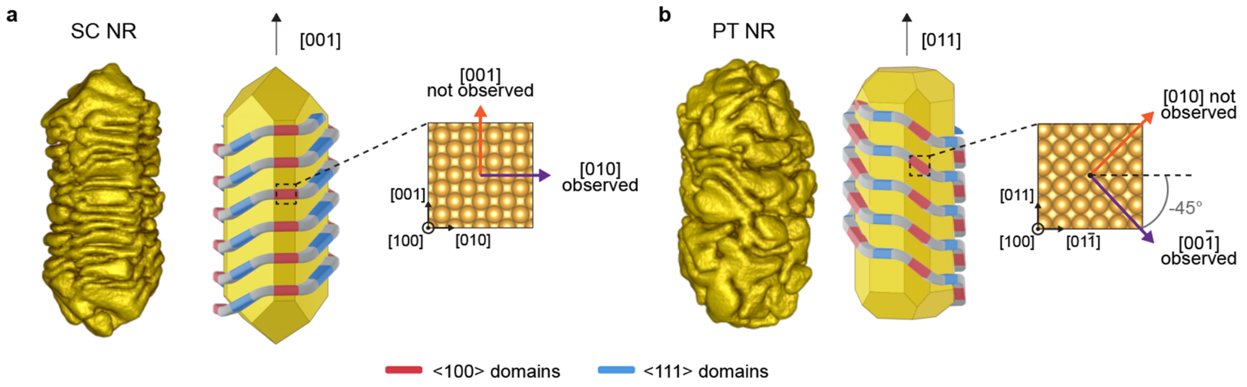

Figure S7. Crystal orientations at the surface of wrinkled Au NRs. **a**, Wrinkles on SC seeds show alignment along the <100> directions which are perpendicular to the long axis of the particle, for example along the [010] direction but rarely along the [001] one on a (100) facet. **b**, Wrinkles grown on PT seeds in presence of S-BINAMINE show alignment along <100> directions at -45° inclination (following convention in **Figure 2** from the main text), e.g., along the [00-1] direction but rarely along the [010] on a (100) facet. In the drawings, areas aligned along <100> crystal directions are in red, areas aligned along <111> are in blue. Note that the models are idealized. Differences with real particles include the fact that PT wrinkles grown in *S*-BINAMINE are mostly observed with negative helical inclination, but occasionally with positive inclination too (**Figure S6**). Nevertheless, the fact that their helicity is strongly negative (**Figure 2**) shows that most wrinkles are indeed with negative inclination.

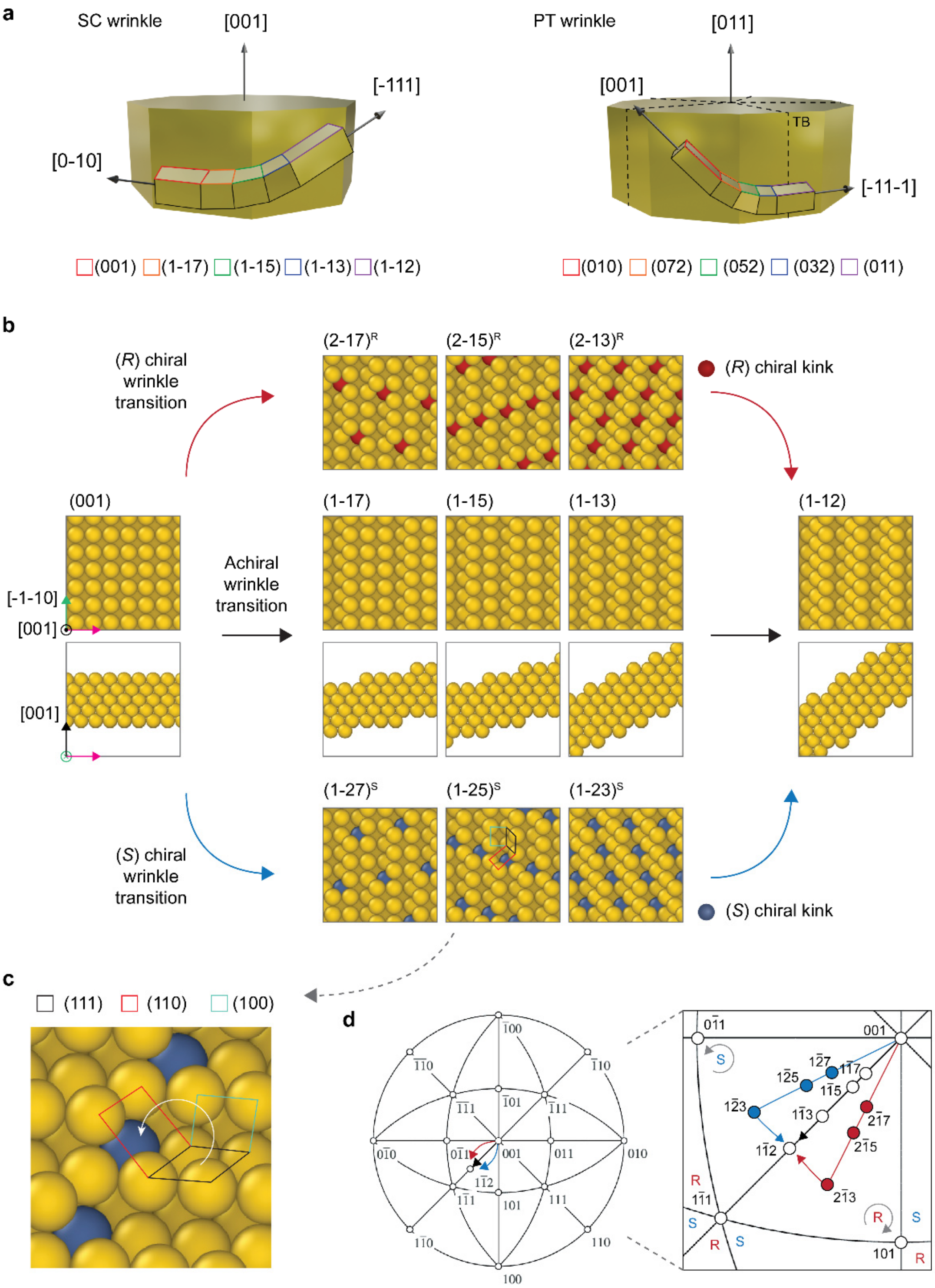

Figure S8. Possible high index (chiral) surfaces in micelle-templated wrinkles.

a) In both SC and PT NR models (here from products obtained in presence of *S*-BINAMINE), the flat → tilted transitions set the *M* or *P* helical chirality of the NR. These morphological transitions correspond to atomic scale transitions. For example, an ideal SC wrinkle goes from exposing (001) planes to exposing (1-12) planes (left). Since the transitions are not atomically sharp (**Figure 3, Figure S4**), we can reasonably assume that they host higher index microfacets or kinks. For example, a smooth in-plane transition in the depicted SC wrinkle would expose (1-17), (1-15) and (1-13) facets. Some of these planes could easily evolve into chiral microfacets or kinks (for example upon deposition of a small amount of Au during growth).[13] A hypothesis is then that the *M* or *P* handedness selection would be

related to the preferential expression of *S*- or *R*-type chiral microfacets in these transitions, similar to the enantioselective development of chiral facets promoted by amino acids in helicoid or twisted NR growth.[14–16]

b) Foregoing chemical considerations about the interactions of axially chiral molecules such as BINAMINE with Au surfaces, this hypothesis can be refuted on geometrical grounds alone. For conciseness, we focus on the SC wrinkle case and start by considering the achiral transition (001) → (1-12) which likely hosts (1-17), (1-15) and (1-13) planes. These planes could easily evolve into the chiral {2-1*l*} or {1-2*l*}, $l = 3, 5, 7$ because their surface normals are close (< 15° of interfacial angle).

Chiral surfaces have normals that do not lie in a mirror plane of the bulk crystal lattice;[13,17] or equivalently, the Miller indices of these {*hkl*} planes obey $h \neq k \neq l \neq h$ and $h \times k \times l \neq 0$. The handedness of such surfaces can be obtained in two ways, which yield identical results for *fcc* metals:

c) Considering that the kinks on a high-index chiral surface are a combination of low-index, (111), (100) and (110) microfacets, it is conventional to attribute the (*S*) or (*R*) handedness based on their arrangement in order of increasing atomic density around the kink. Here, the (111) > (100) > (110) arrangement is counterclockwise around the kinks in the (1-25) facet; the facet is therefore of the (*S*) handedness.

d) Equivalently, the handedness can be determined from the stereographic projection of *fcc* normals. Chiral surfaces are those with their normal direction not lying in a mirror plane of the *fcc* lattice, and all those within the same "stereographic triangle" bounded by three mirror zones share the same handedness.[17] The absolute handedness can be determined from the arrangement of poles around a stereographic triangle in order of increasing symmetry, i.e., <110> (two mirrors), <111> (three mirrors), <100> (four mirrors), for the *fcc* lattice.[17]

Thus, the $\{2\text{-}1l\}^R$ and $\{1\text{-}2l\}^S$ planes are confirmed to be chiral and of opposite handedness, and there are two (*S*)-chiral and (*R*)-chiral pathways for the (001) → (1-12) transition that are similarly close to the achiral pathway (**b**, **d**). In other words, a *P* helical pattern on a SC NR could be obtained with either *R*- or *S*-chiral transitions. Therefore, the atomic-scale hypothesis cannot explain the handedness selection at the NR level.

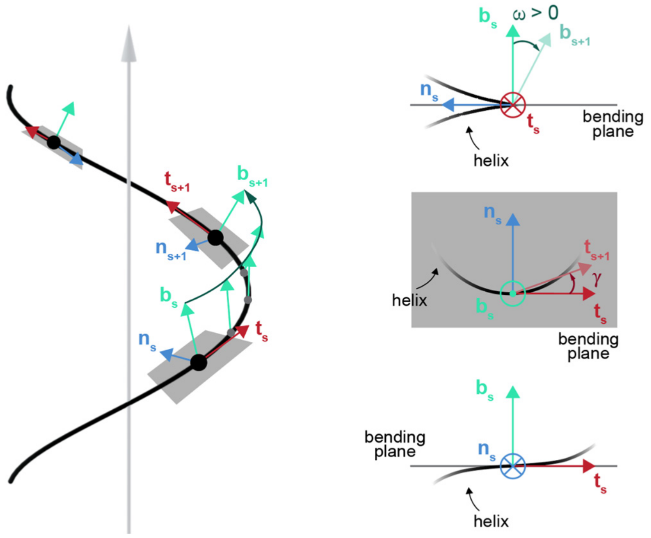

$$\tau = \frac{\omega}{\Delta s}$$

$$\kappa = \frac{\gamma}{\Delta s}$$

Figure S9. Torsion and curvature of helices. The torsion τ and curvature κ of a space curve (black line) can be computed from the evolution of the Frenet-Serret frame along its arc-length s. The frame is described at each point of the curve (black and gray dots) by three orthogonal unit-vectors: a tangent vector **t**(s) (red), a normal vector **n**(s) (blue), and a binormal vector **b**(s) (cyan). κ is defined as the speed of rotation of **t** along the curve. Between two arc-length positions (s $\rightarrow$ s+1), the plane in which the tangent rotates by γ is the bending plane. **n**(s) is perpendicular to the tangent and lies in this plane. **b**(s) is perpendicular to the plane. τ is the rate at which the bending plane (the binomial vector) rotates. For a right-handed *P*-helix as drawn here, the angle ω of the rotation of **b**(s $\rightarrow$ s+1) is constant and by convention, positive. The torsion is therefore constant and positive along its arc-length.

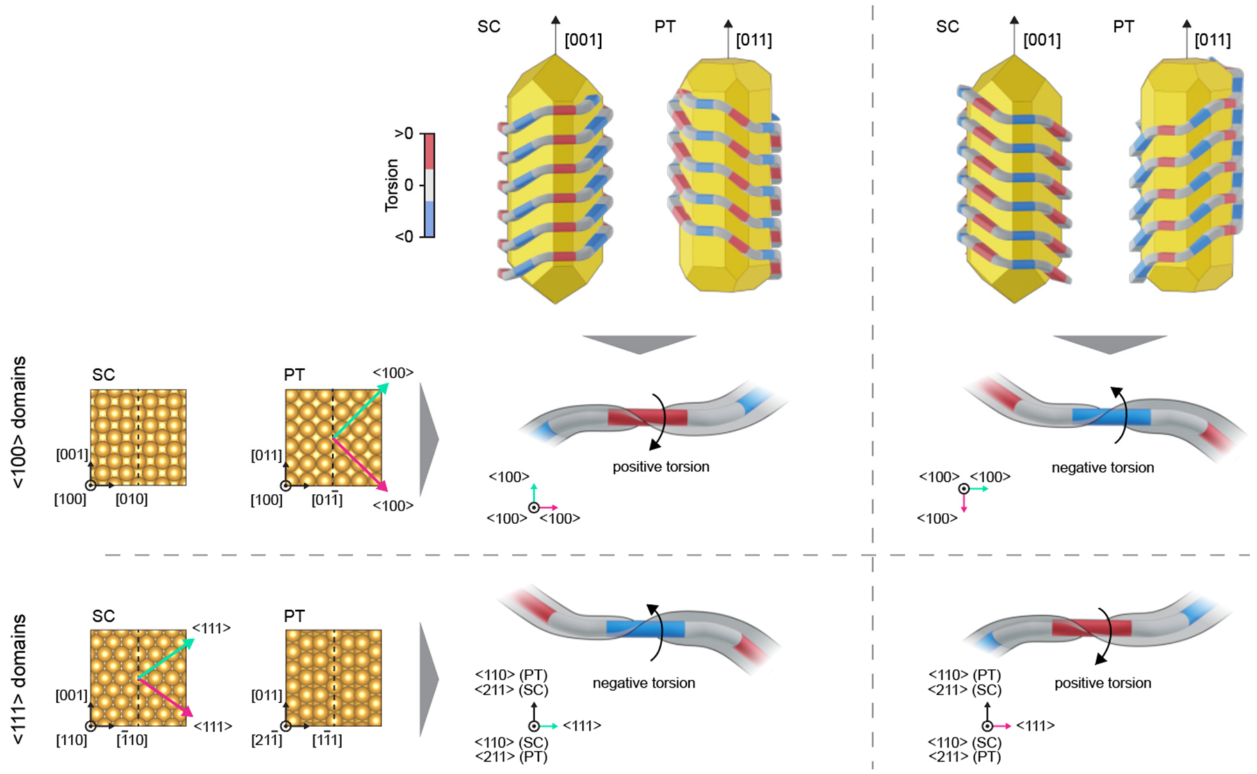

Figure S10. Comparison of wrinkle torsion in NRs grown with *S*- or *R*-BINAMINE. Double entry table showing the wrinkle torsion in micelle-templated NRs as a function of the molecular inducer enantiomer (columns) and the alignment direction (rows). The case of *S*-BINAMINE (first column) is as presented in **Figure 4**; on either SC and PT NRs, positive wrinkle torsion is observed along <100> crystal directions (first row), negative wrinkle torsion is observed along <111> directions (second row). For NRs grown in presence of *R*-BINAMINE (second column), the patterns are reversed; on either seeds, positive wrinkle torsion is observed along <111> crystal directions (second row), negative wrinkle torsion is observed along <100> (first row). This reversal is permitted by the presence of equivalent crystal directions compatible with the alignment of wrinkles (cyan and magenta arrows). The choice of BINAMINE enantiomer determines the selection of alignment. The choice of seed determines along which family (<100> or <111>) this selection is done. Thus, it is the combination of the crystal surface of the seed and of the BINAMINE enantiomer that determines the helical chirality. Note that the red/blue color scale corresponds to the torsion sign in this figure.

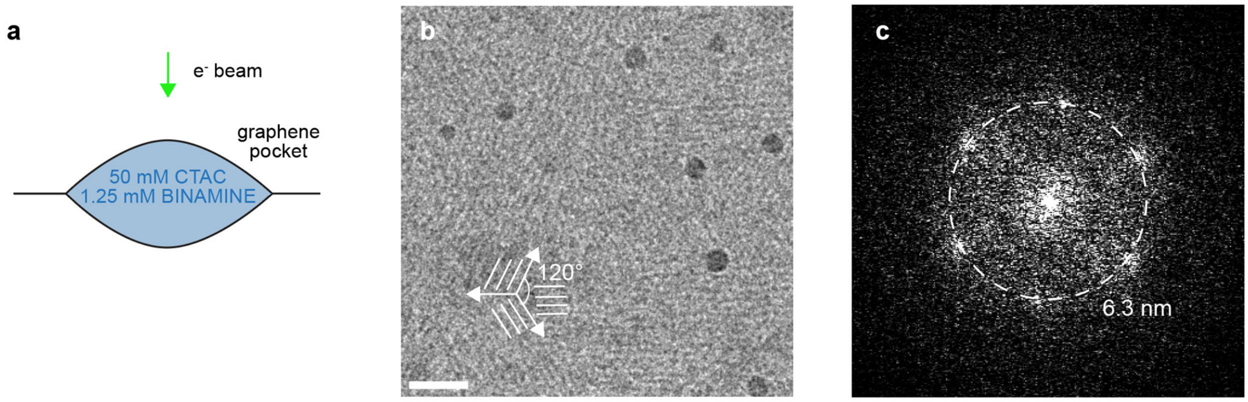

Figure S11. Orientational order of BINAMINE-CTAC multimicellar assemblies in graphene liquid cells (GLC). **a**, Depiction of the GLC experimental setup: the growth solution (50 mM CTAC, 1.25 mM BINAMINE) was enclosed between two graphene layers and imaged in TEM. **b**, HRTEM image of a graphene liquid pocket containing the growth solution, **c**, corresponding FFT. White lines are guides to the eye showing the typical spacing of the features. Low contrast structures were observed to have a 3-fold symmetry and a typically ~ 6.3 nm period which is consistent with that of CTAC assemblies reported on graphite surfaces.[4,5,18] The darker, spherical features are likely Au or Cu, which is a typical contamination of the GLC fabrication process.[19] The scale bar is 50 nm.

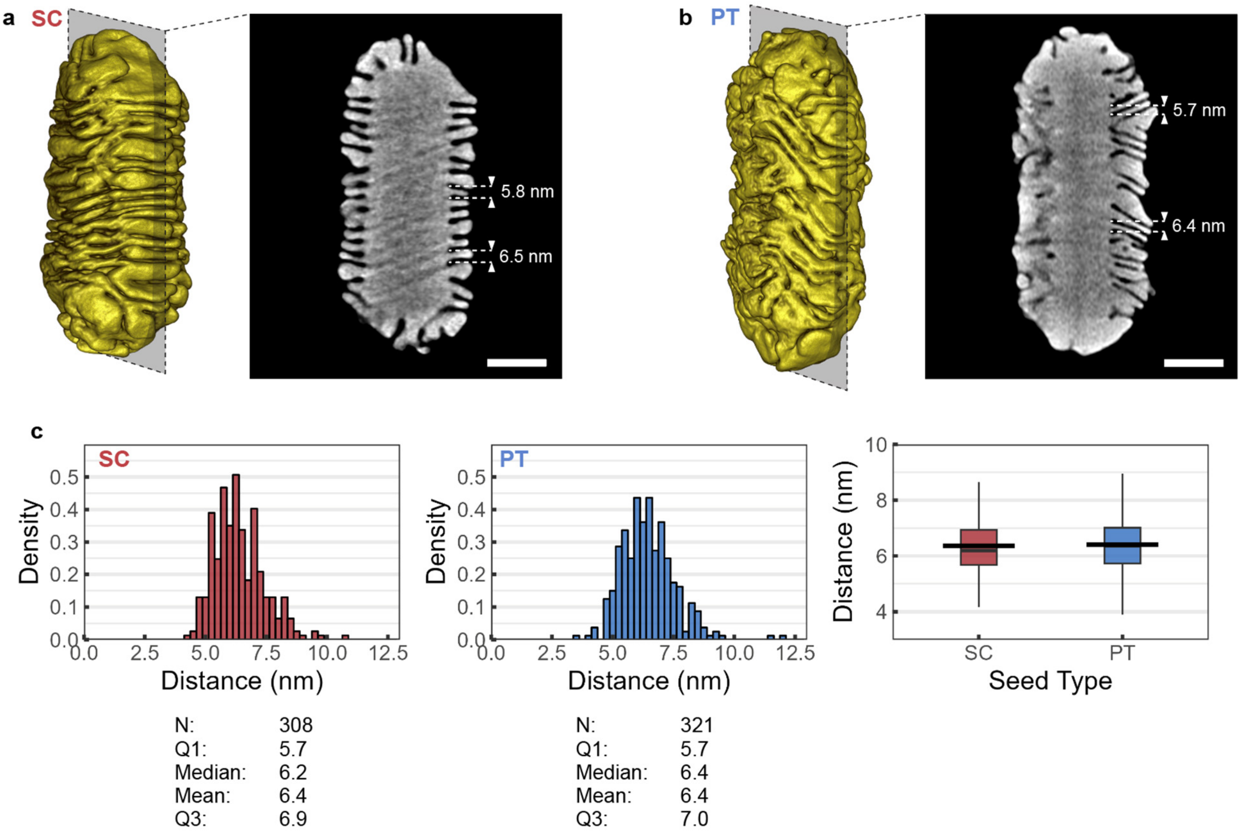

Figure S12. Distribution of the groove-to-groove distance on Au NRs. **a**, Isosurface rendering (left) and slice along the shaded plane (right) from electron tomography reconstructions of a typical SC wrinkled NR and, **b**, a PT wrinkled NR, both grown in presence of *S*-BINAMINE. Typical groove-to-groove distances between wrinkles are shown in the slices. The scale bars are 40 nm. **c**, Histograms and box plots of the groove-to-groove distances across 6 SC NRs and 11 PT NRs, totaling N > 300 measurements for each particle type. The box plots show the quartiles, whiskers are at most 1.5 x the interquartile range, black crossbars show the mean. The descriptive statistics are summarized below the histograms. Measurements were done manually in areas where the wrinkles appeared ordered, as typically shown in panels a and b. These areas are ubiquitous in any particles analyzed herein, but not unique. Some intra- and inter-particle polydispersity is typically observed, as shown in **Figure S4**, suggesting that the long-range order of the templating micelles can easily be influenced. Overall the groove-to-groove distance appears very close to the inter-micellar distance measured on ordered assemblies of CTAC molecules on Au(111).[3]

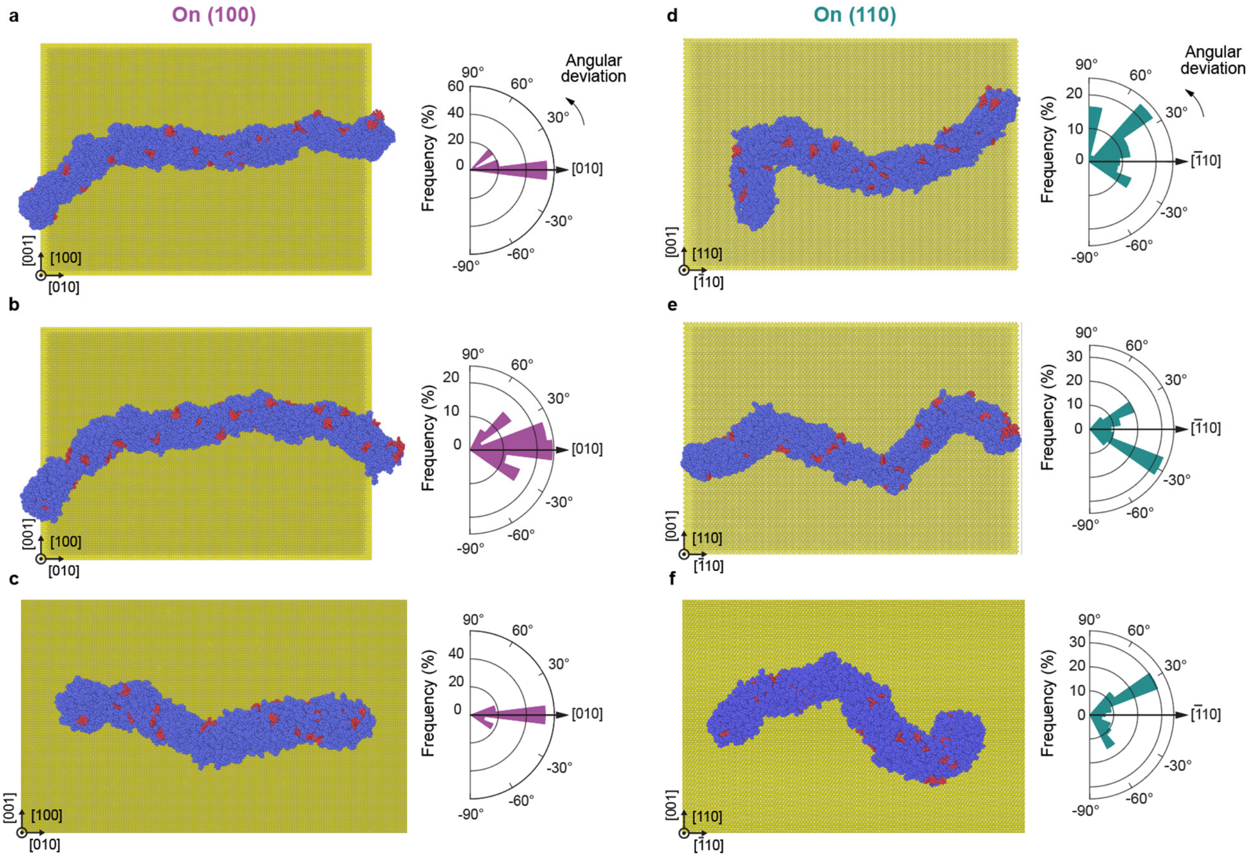

Figure S13. MD simulations of BINAMINE-CTA$^+$ assemblies on Au. **a-c**, Simulations of a BINAMINE (red) – CTA$^+$ (blue) assembly on Au(100) (left) and resulting polar histograms of the angular deviation (right) from the long axis of the substrate. The long axis of the substrate corresponds to a <100> direction. The histograms were obtained for N = 1000 small elements along the fitted center line of each micelle (**Figure S14c**). **d-e**, Simulations on Au(110) (left) and corresponding polar histograms of the angular deviation (right). The long axis of the substrate corresponds to a <110> direction.

Simulations shown in **a, b, d** and **e** were obtained with *R*-BINAMINE, simulations in **c** and **f** were with *S*-BINAMINE. The *R*-BINAMINE simulations used a 300 Å-long substrate, which was shorter than the micelle and likely produced edge effects. Substrates for the simulations with *S*-BINAMINE were increased to 600 Å to prevent this. Nonetheless, the discrepancy in preferential alignment was reproduced with either enantiomer and on both short and long substrates: histograms peaked around 0° for micelles on Au(100), and around ± 30° on Au(110).

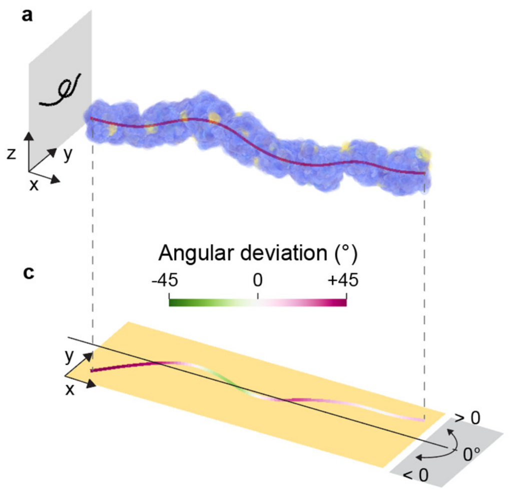

Figure S14. Geometric properties of micelles from MD simulations. a, the centerline (red) of the micelle was retrieved by fitting a spline to the atomic position obtained from the simulations. The gray plane depicts the projection (black line) of the centerline along the ***x*** direction and onto the *zy* plane of the simulation frame. **b**, From the evolution of the Frenet-Serret frame along the centerline (**Figure S9**), we obtained the torsion (rotation of the bending plane along the arc-length of the line) and, **d**, the curvature (rotation of the tangent vector along the arc-length of the line). **c,** In addition, we computed the in-plane angular deviation with respect to a given direction. This direction was typically the ***x*** direction (black line), which corresponded to a <100> or a <110> Au crystal direction in the simulations.

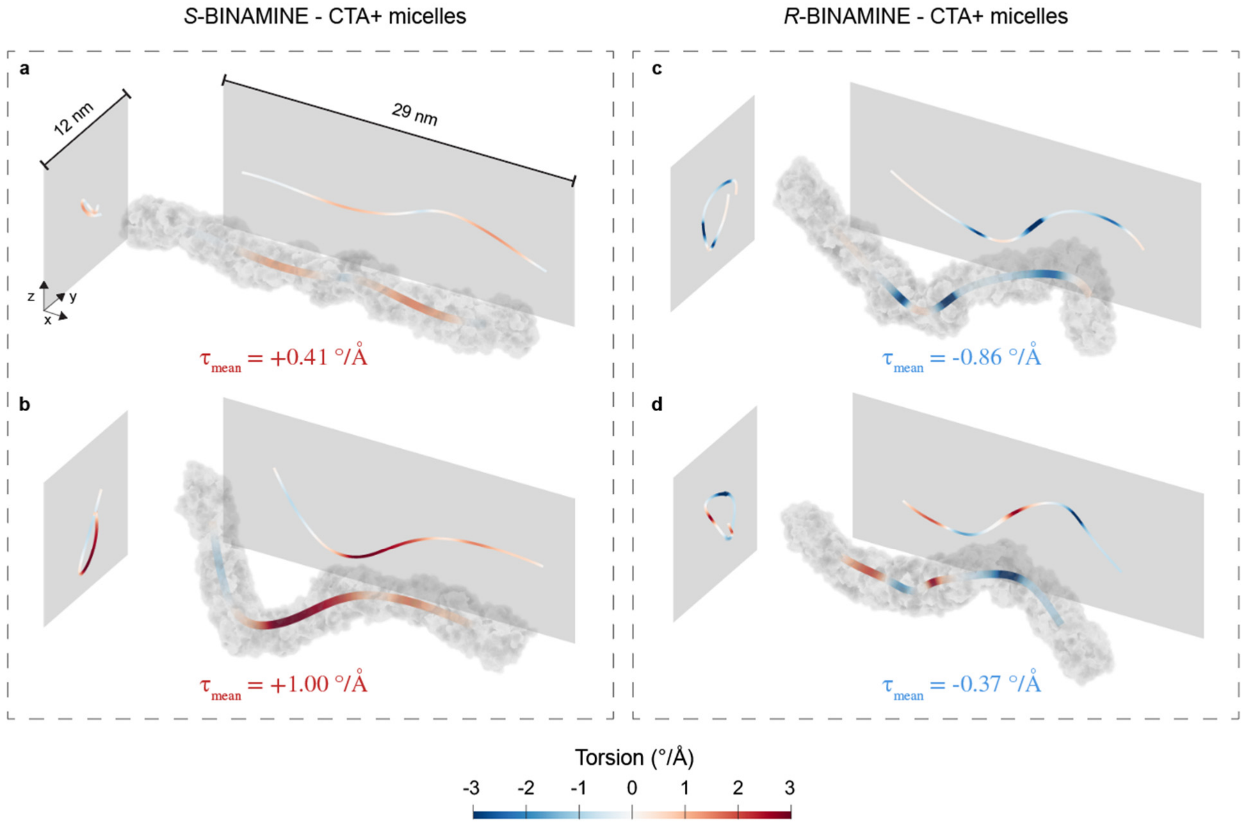

Figure S15. MD simulations of isolated BINAMINE-CTA$^+$ assemblies. **a, b**, simulations of *S*-BINAMINE-CTA+ and **c, d**, *R*-BINAMINE-CTA+ assemblies (gray) in vacuum. The torsion was computed form the rotation of the Frenet-Serret frame along the fitted center line (red-blue line) of each stabilized micelle. Gray planes depict projections of the color-coded centerline along the x and y axis of the simulation frame. Areas with varying torsion coexisted in all micelles, but those with *S*-BINAMINE typically had dominant positive torsion as seen by the positive mean torsion along the center line, those with *R*-BINAMINE had dominant negative torsion.

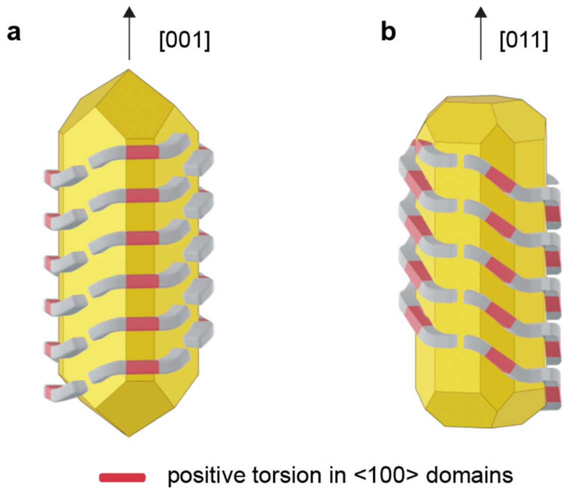

Figure S16. Discontinuous models of wrinkles and adsorbed micelles. **a**, Idealized model of the wrinkle pattern on the SC and, **b**, PT intermediates grown in presence of *S*-BINAMINE in a hypothetical discontinuous case. Unlike the continuous model (main **Figure 4**), which requires periodic domains of negative and positive torsion, the discontinuities allow building *M* and *P*-helical patterns with units of positive torsion only. The wrinkle pattern is likely templated by a corresponding pattern of micelles adsorbed on the seeds. As such, it could arise from the scission of the micelles in the <111> domains, possibly under the combined action of alignment torque and BINAMINE-induced torsion.

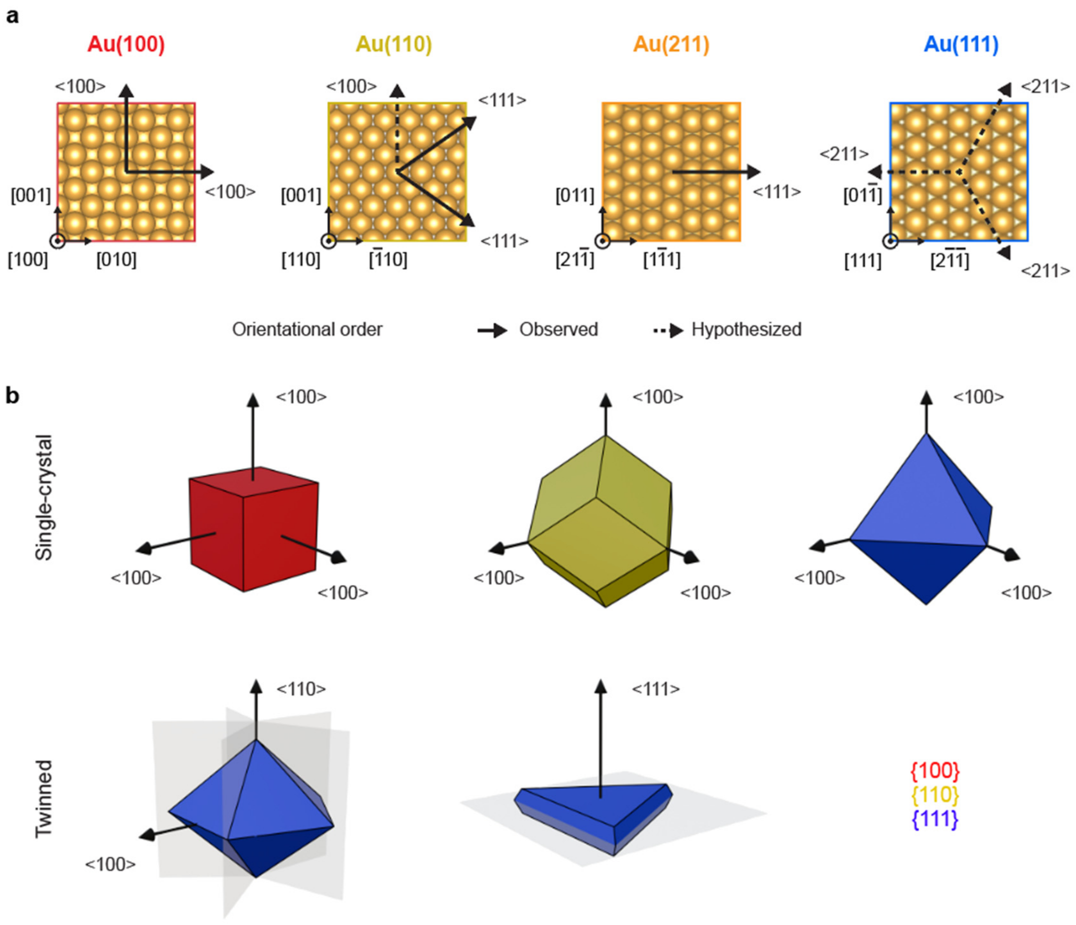

Figure S17: Potential for orientational order of CTAC-BINAMINE micelles on common Au surfaces and seeds. **a**, Low index Au surfaces and crystal directions along which orientational order has been observed in this work (solid arrows) or is hypothesized based on this work and the literature[2] (dashed arrows). **b**, Common Au nanoparticles used as seeds, including single-crystal cube,[16] rhombic dodecahedron,[20] octahedron,[21] and twinned (shaded plane) decahedron[22] or prism.[23] The facets are color-coded based on the exposed crystal planes, which sets the orientational order of micelles. It should be noted that since these seeds lack a major axis, the transfer from the chiral selection rule at the wrinkle scale (i.e., for S-BINAMINE that wrinkle torsion is positive along <100> and negative along <111>) to the macroscale is an open question. Indeed, without a major axis to guide the arrangement of chiral centers, their effects may cancel out the chiroptical response.[22] Furthermore, the exposed facets may be subject to change in the early stages of growth (see **Figure S2**).

Supplementary Tables

Table S1. Overview of NR synthesis. The time column indicates the time at which the synthesis was stopped when the NR was part of a kinetic series. If not applicable (N.A.), the reaction was conducted until exhaustion of the reactants, typically for about 30 minutes.

<table>
<tr><th>Seed</th><th>BINAMINE</th><th>Time (s)</th><th>Seed size (l x d, nm)</th><th>Reference for synthesis</th><th>Figure</th></tr>
<tr><td rowspan="8">SC</td><td rowspan="6">S</td><td>5</td><td rowspan="5">104 x 25</td><td rowspan="5">[12]</td><td>1, S2a</td></tr>
<tr><td>30</td><td>1, S5</td></tr>
<tr><td>60</td><td>1, 2a, 2d, 2e, 2g, S3, S10</td></tr>
<tr><td>120</td><td>2d, 2g, S10</td></tr>
<tr><td>300</td><td>2d, 2g, S10</td></tr>
<tr><td>N.A.</td><td>107 x 27</td><td>[24]</td><td>2d, 2g</td></tr>
<tr><td rowspan="2">R</td><td>5</td><td rowspan="2">104 x 25</td><td rowspan="2">[12]</td><td>S1</td></tr>
<tr><td>30</td><td>3</td></tr>
<tr><td rowspan="8">PT</td><td rowspan="7">S</td><td>30</td><td rowspan="4">82x 20</td><td rowspan="4">[12]</td><td>1, S5</td></tr>
<tr><td>60</td><td>1, 2b, 2d, 2e, 2g, S3, S10</td></tr>
<tr><td>120</td><td>2d, 2g, S10</td></tr>
<tr><td>300</td><td>2d, 2g, S10</td></tr>
<tr><td>N.A.</td><td>130 x 32</td><td>[24]</td><td>2d, 2g, S10</td></tr>
<tr><td>N.A.</td><td>138 x 25</td><td>[25]</td><td>2d, 2g, S10</td></tr>
<tr><td>N.A.</td><td>138 x 30</td><td>[25]</td><td>S10</td></tr>
<tr><td>R</td><td>20</td><td>82 x 20</td><td>[12]</td><td>S2b, S4, S2</td></tr>
</table>

Table S2. Morphological measurements of wrinkles. Wrinkles were grown on SC or PT seeds in presence of S-BINAMINE. The measurements were conducted following the methods described in **Figure S3**. 10,000 measurement points per NR were sampled, for N = 2 NRs of each type.

	Wrinkle thickness (nm) Random sampling				Wrinkle thickness (nm) Volume-corrected sampling				Groove thickness (nm) Random sampling			
	mean	median	IQR	mode	mean	median	IQR	mode	mean	median	IQR	mode
SC60	9.43	7.66	6.04	5.97	5.88	5.29	2.7	3.47	2.56	2.22	1.11	2.12
PT60	9.79	9.36	4.85	8.94	7.26	6.77	4.24	4.2	2.91	2.68	1.39	2.37

Supplementary References

1. Jaschke, M., Butt, H.-J., Gaub, H. E. & Manne, S. Surfactant Aggregates at a Metal Surface. *Langmuir* **13**, 1381–1384 (1997).
2. Schniepp, H. C., Shum, H. C., Saville, D. A. & Aksay, I. A. Orientational Order of Molecular Assemblies on Rough Surfaces. *J. Phys. Chem. C* **112**, 14902–14906 (2008).
3. Schniepp, H. C., Saville, D. A. & Aksay, I. A. Tip-Induced Orientational Order of Surfactant Micelles on Gold. *Langmuir* **24**, 626–631 (2008).
4. Saville, D. A. *et al.* Orientational Order of Molecular Assemblies on Inorganic Crystals. *Phys. Rev. Lett.* **96**, 018301 (2006).
5. Chun, J., Li, J.-L., Car, R., Aksay, I. A. & Saville, D. A. Anisotropic Adsorption of Molecular Assemblies on Crystalline Surfaces. *J. Phys. Chem. B* **110**, 16624–16632 (2006).
6. Li, J.-L. *et al.* Use of dielectric functions in the theory of dispersion forces. *Phys. Rev. B* **71**, 235412 (2005).
7. Manne, S. & Gaub, H. E. Molecular Organization of Surfactants at Solid-Liquid Interfaces. *Science* **270**, 1480–1482 (1995).
8. Asgari, M. A molecular model for the free energy, bending elasticity, and persistence length of wormlike micelles. *Eur. Phys. J. E* **38**, 98 (2015).
9. Cates, M. E. & Candau, S. J. Statics and dynamics of worm-like surfactant micelles. *J. Phys.: Condens. Matter* **2**, 6869–6892 (1990).
10. Smith, J. D. *et al.* Defect-Directed Growth of Symmetrically Branched Metal Nanocrystals. *Angew. Chem. Int. Ed.* **59**, 943–950 (2020).
11. Midgley, P. A. & Weyland, M. 3D electron microscopy in the physical sciences: the development of Z-contrast and EFTEM tomography. *Ultramicroscopy* **96**, 413–431 (2003).
12. Van Gordon, K., Girod, R., Bevilacqua, F., Bals, S. & Liz-Marzán, L. M. Structural and Optical Characterization of Reaction Intermediates during Fast Chiral Nanoparticle Growth. *Nano Lett.* **25**, 2887–2893 (2025).
13. Shukla, N. & Gellman, A. J. Chiral metal surfaces for enantioselective processes. *Nat. Mater.* **19**, 939–945 (2020).
14. Ni, B. *et al.* Chiral Seeded Growth of Gold Nanorods Into Fourfold Twisted Nanoparticles with Plasmonic Optical Activity. *Adv. Mater.* **35**, 2208299 (2023).
15. Im, S. W. *et al.* Investigating chiral morphogenesis of gold using generative cellular automata. *Nat. Mater.* **23**, 977–983 (2024).
16. Lee, H.-E. *et al.* Amino-acid- and peptide-directed synthesis of chiral plasmonic gold nanoparticles. *Nature* **556**, 360–365 (2018).
17. Jenkins, S. J. & Pratt, S. J. Beyond the surface atlas: A roadmap and gazetteer for surface symmetry and structure. *Surface Science Reports* **62**, 373–429 (2007).
18. Aksay, I. A. *et al.* Biomimetic Pathways for Assembling Inorganic Thin Films. *Science* **273**, 892–898 (1996).
19. Pedrazo-Tardajos, A. *et al.* Direct visualization of ligands on gold nanoparticles in a liquid environment. *Nat. Chem.* **16**, 1278–1285 (2024).
20. Lee, H.-E. *et al.* Cysteine-encoded chirality evolution in plasmonic rhombic dodecahedral gold nanoparticles. *Nat Commun* **11**, 263 (2020).
21. Cho, N. H. *et al.* Uniform Chiral Gap Synthesis for High Dissymmetry Factor in Single Plasmonic Gold Nanoparticle. *ACS Nano* **14**, 3595–3602 (2020).
22. Sun, X. *et al.* Tuning the Geometry and Optical Chirality of Pentatwinned Au Nanoparticles with 5-Fold Rotational Symmetry. *ACS Nano* **18**, 9543–9556 (2024).
23. Xu, L. *et al.* Enantiomer-dependent immunological response to chiral nanoparticles. *Nature* **601**, 366–373 (2022).
24. Van Gordon, K. *et al.* Single Crystal and Pentatwinned Gold Nanorods Result in Chiral Nanocrystals with Reverse Handedness. *Angew. Chem. Int. Ed.* **63**, e202403116 (2024).
25. Bevilacqua, F., Liz-Marzán, L. M., Bevilacqua, F. & Liz-Marzán, L. M. Seeded Growth of Large Gold Nanorods Modulated by Halide-Mediated Kinetics. *MI* **2**, 388–396 (2025).
